# Nitrones: Comprehensive Review on Synthesis and Applications

**DOI:** 10.3390/molecules31010013

**Published:** 2025-12-19

**Authors:** Ricardo A. L. S. Santos, Artur M. S. Silva, Diana C. G. A. Pinto

**Affiliations:** LAQV-REQUIMTE, Department of Chemistry, University of Aveiro, 3010-193 Aveiro, Portugal; ricardossantos@ua.pt (R.A.L.S.S.); artur.silva@ua.pt (A.M.S.S.)

**Keywords:** nitrone, organic synthesis, oxidation, cycloaddition, spin-trap, therapeutics, polymers

## Abstract

Nitrones are a significant class of compounds highly useful in organic synthesis; in particular, they are key intermediates for the synthesis of new biologically active nitrogen compounds. The first part of this review aims to provide a structured and concise overview of nitrones, summarizing their synthetic methodologies and highlighting the environmentally friendly approaches. Their fundamental transformations and a thorough explanation of their reactivities are addressed, either in rearrangements to similar compounds or as fragments/intermediates for more complex molecules. Lastly, physicochemical properties, therapeutic potential, and industrial applications are also addressed. This review gives an update on the scientific discoveries in the field of nitrones, focusing on organizing the existing information and highlighting subtle details to enhance chemical comprehension.

## 1. Introduction

**Figure 1:**
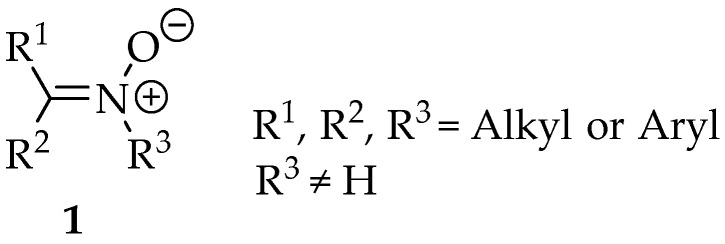
Nitrone’s structure.

The nitrones (Figure 1) are a family of organic compounds known since 1890, when they were first synthesized by Beckmann [1]. Since then, lots of scientific material has been published and research into them is intense. They were first named by Pfeiffer in 1916, due to their similarity with ketones, in terms of reactivity [2]. Then, they became famous for their good enantioselectivity in 1,3-dipolar cycloadditions, and nowadays there is a huge repertoire of syntheses where they can be implemented as intermediates. Furthermore, the nitrones themselves can be applied as spin traps, and they have anti-oxidative properties, useful in the production of polymers and in biological systems [3].

**Figure 2:**
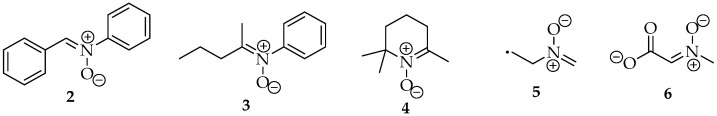
Five nitrones’ examples: (*Z*)-*N*,1-diphenylmethanimine oxide **2**; (*Z*)-*N*-phenylpentan-2-imine oxide **3**; 2,2,6 trimethyl-2,3,4,5 tetrahydropyridine oxide **4**; 2-(methylenoxo-λ^5^-azanyl)ethyl **5;** and (*Z*)-2-(methyloxo-λ^5^-azanylidene)ethanoate **6**.

In early days, the nitrones were named according to their family’s name, with the substituents allocated to their α- or *N*- positions. For example, nitrone **2** (Figure 2) was named *N*,α diphenylnitrone **2** [4]. However, this nomenclature is no longer recommended by IUPAC, according to the rules in the blue book of 2013 [5]. It is even misleading, as the α-carbon should not be the sp^2^ carbon bound to the nitrogen, but the one next to it, like in ketones. The correct name, or the preferred IUPAC name (PIN), is now (*Z*)-*N*,1-diphenylmethanimine oxide **2**. They are named after their imine analog, followed by the term oxide. For the structures **3** and **4**, their names are (*Z*)-*N*-phenylpentan-2-imine oxide and 2,2,6 trimethyl 2,3,4,5 tetrahydropyridine oxide, respectively.

In terms of priority among other compound classes, following IUPAC rules, they rank fifth because they are zwitterions [5]. Therefore, this group is named as substituent only when there are radicals or anions in the same structure. In those cases, the expression “oxo-λ^5^-azanyl” is used. For example, in structure **5**, the preferred name should be 2-(methylenoxo-λ^5^-azanyl)ethyl, whereas for structure **6**, the suffix -idene must be added, as the nitrone group is linked to the main chain by the double bond, so the name is (*Z*)-2-(methyloxo-λ^5^-azanylidene)ethanoate. When naming these compounds, one should not forget the diastereoisomerism of the double bond. Usually, they are more stable in the *Z* configuration due to steric effects from the substituents.

**Scheme 1:**
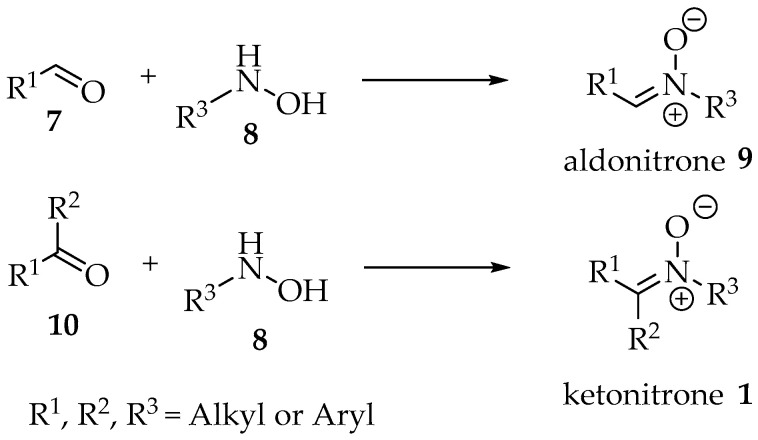
Reaction between hydroxylamine derivatives **8** and aldehydes **7** or ketones **10** as justification for the terms aldonitrone **9** and ketonitrone **1**.

Finally, one distinction often made is between aldonitrones and ketonitrones (Scheme 1) [4]. In the first case, only one substituent is present in the sp^2^ carbon, whereas in the second case the nitrone is fully substituted. These designations should result from the reaction between hydroxylamine derivatives and aldehydes or ketones, respectively (Scheme 1).

Some less recent reviews focus on the reactions of nitrones, particularly 1,3-dipolar cycloadditions and the synthesis of heterocyclic compounds [2,3,4,6,7,8,9,10], and the newest one is dedicated to describing the results of a single research group. This review aims to summarize that content while emphasizing mechanistic details, including the latest developments. It also clearly organizes the key aspects of nitrone chemistry. Additionally, there is a dedicated section discussing the applications of nitrones beyond organic synthesis.

## 2. Nitrone Synthesis

**Figure 3:**
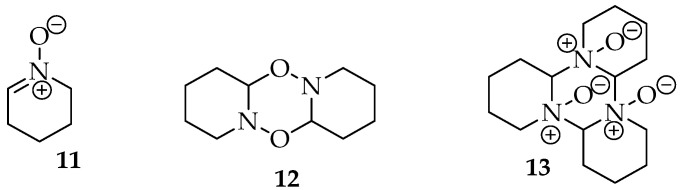
Dimer **12** and trimer **13** of 2,3,4,5 tetrahydropyridine oxide **11**, through C-O and C-N bonding, respectively [4].

Various methods have been employed to prepare nitrones, which are hygroscopic products. These products can exist as oils, typically for aliphatic structures, or as solid compounds, generally for aromatic structures, and they have a transparent or white appearance for simpler products [11,12]. In the first case, there is even a trend for the nitrones to form dimers, though structures with higher complexity can also be formed (Figure 3), which can complicate the obtention of the desired compound [4,11].

### 2.1. Oxidations

**Scheme 2:**
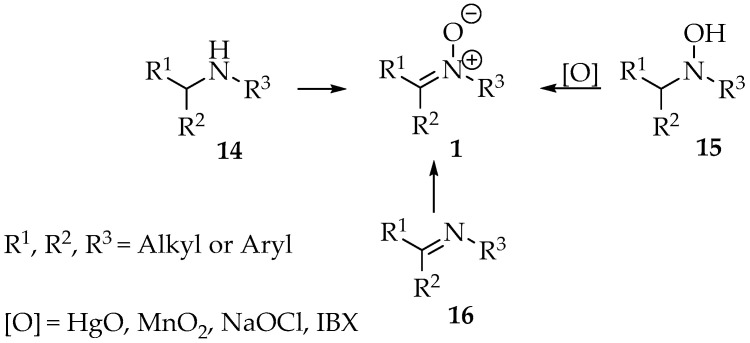
Nitrones **1** formation by oxidation of secondary amines **14**, *N*,*N* dialkyl hydroxylamines **15** and imines **16**.

One approach involves the oxidation of nitrogenous precursors, such as secondary amines, hydroxylamines, and imines (Scheme 2), with the use of hydroxylamines being the least used method due to the use of highly toxic reagents, such as mercuric oxide [3]. Nevertheless, a more environmentally friendly approach using sodium hypochlorite was successfully tested [13]. In the early days, lead and mercury oxides and potassium permanganate were commonly used as oxidizing agents, but an urge to lower the ecological footprint has led to the development of reactions using hydroperoxides or air’s oxygen, sometimes in the presence of catalysts [4].

In the case of hydroxylamine derivatives **15**, various oxidants have been reported in the literature. Examples include *t*-butyl hydroperoxide, *m*-CPBA (3-chlorobenzene-1-carboperoxoic acid), airflow (catalyzed by Cu^2+^), and both palladium and mercury(II) oxide (Scheme 2) [3,12,13,14,15]. Although this method is not widely used, Goti and colleagues have developed several approaches that operate under mild conditions, primarily utilizing less hazardous catalytic systems [13,16,17,18,19]. It is worth highlighting the efficient use of poly(4-vinylpyridine)/MTO compounds or microencapsulated polystyrene/MTO systems as catalysts, which could be recovered and used with hydrogen peroxide, making this procedure an environmentally friendly one (Scheme 2) [19].

**Scheme 3:**
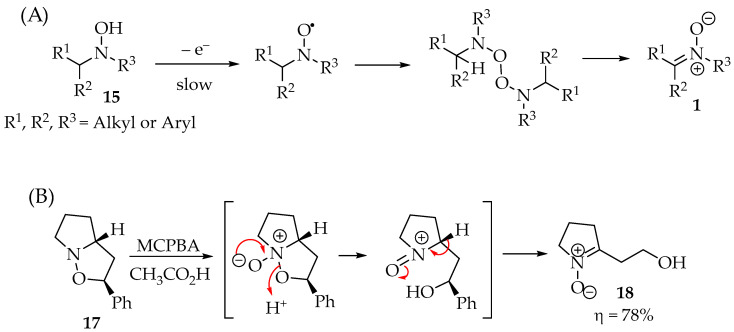
(**A**) Hydroxylamine **15** oxidation to nitrone **1**; (**B**) isoxazolidine **17** oxidation into nitrone **18** and the respective mechanism.

To better elucidate the redox chemistry phenomena in these reactions, Scheme 3A shows the process’s main steps. Although they are not exclusive, as the reactions depend on the oxidant reagents used, they are the most ubiquitous for hydroxylamine derivatives **15**. In the case of the hydroxylamine derivatives **15**, they lose the *N*-hydroxyl hydrogen, forming the nitroxide radical. The simplicity of this step determines the hydroxylamines’ anti-oxidative properties in their applications. Then, depending on the substituents’ hindrance, they may either form a dimer or proceed readily to their disproportionation. As a result, the nitrone product **1** is obtained from each of the two nitroxide radicals, and the hydroxylamine reagent **15** can be recovered (Scheme 3A) [20]. An illustrative example is the oxidation of isoxazolidine **17** (Scheme 3B) [21,22], whereas the isoxazolidine’s behavior is similar to that of hydroxylamine.

Although isoxazolidine are heterocyclic structures commonly synthesized from nitrones, they can form new nitrones as well. The N-O bond can be cleaved with oxidants, producing γ-hydroxynitrones **18** (Scheme 3B). The reaction starts with the nucleophilic attack of the nitrogen to the oxidant species, such as *m*-CPBA, resulting in the isoxazolidine *N*-oxide. Then, the ring opens, forming a nitrosonium ion derivative that is released from an α-hydron, restores the molecular neutrality, and forms, selectively, the nitrone **18** in very good yields (Scheme 3B).

For amines and imines, DMD (dimethyldioxirane) and Oxone^®^ have also been used [3,12]. Hydrogen peroxide is another option in an aqueous solution or a solid complex with urea. However, a catalyst is required to promote the organic compound oxidation instead of the peroxide disproportionation [23]. Sodium tungstate seems to provide the best results [23]. To guarantee the highest conversion, the oxidant is often present in significant excess, having to be removed/reduced at the reaction end. The photocatalytic oxidation of aldimines to nitrones by the O_2_/TiO_2_ system achieved good yields (>80%) in one study [24]. An efficient metal-free procedure, using only Oxone^®^ as oxidant and a biphasic basic medium composed of acetonitrile and THF (4:1) and an aqueous of EDTA and NaHCO_3_ solution, was used to obtain nitrones from amines [25]. The yields were very good, and most importantly, other functional groups or existing stereogenic centers were tolerant to the conditions used.

**Scheme 4:**
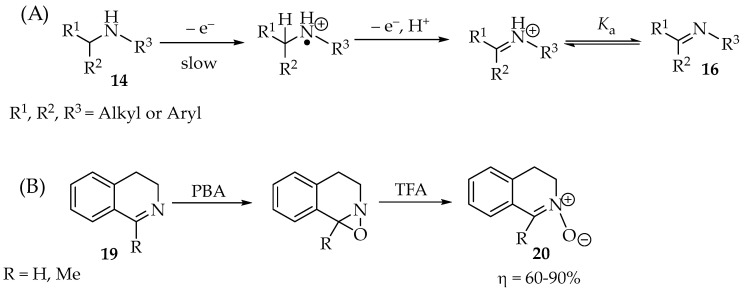
(**A**) Amine **14** oxidation to imine **16**, and (**B**) example with further oxidation to nitrone **20**.

The amines **14** are usually oxidized by losing one of the electrons in the electron pair, forming a radical cation (Scheme 4A). Then, it loses the *α*-hydrogen (or a hydron and an electron, depending on whether the mechanism is concerted or not) to produce the imine **16** [26,27]. In molecules with other *α*-nucleophiles, such as the carboxylic group in *α*-amino acids, they might be lost instead [9].

The imines, such as **19** (Scheme 4B), are more acidic than the corresponding amines. Therefore, the following deionization happens to a good extent. Primary amines require stronger oxidants than secondary or tertiary amines. Their anodic (oxidation) potentials against Ag/0.1 M Ag^+^ (MeCN) are between 1.2 and 2.0 V for primary amines, between 0.82 and 1.08 V for secondary amines, and 0.71 V for the *N*,*N*-dimethylbenzylamine [26,28]. The imine **19** might be further oxidized by the same oxidant, given enough time and quantity of the PBA reagent, to an oxaziridine derivative (Scheme 4B). By acidification of the mixture, for example, with trifluoroacetic acid (TFA), the nitrone **20** is formed in moderate to excellent yields, depending on the solvent used (Scheme 4B). Sometimes, the intermediate compound converts readily to the nitrone without further processing [3,29,30].

### 2.2. Nucleophilic Substitutions

**Scheme 5:**
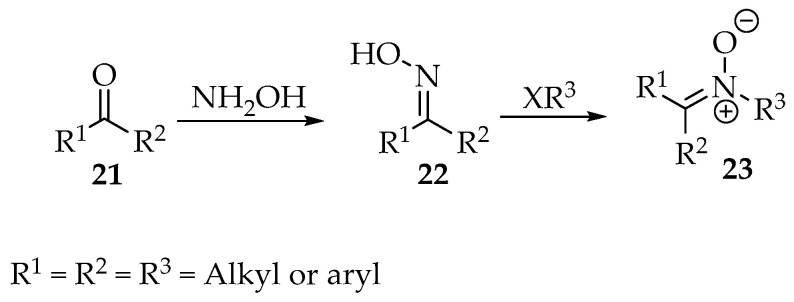
General synthesis of nitrones **23**, through nucleophilic substitutions involving oximes **22**.

Nucleophilic substitutions of carbonyl compounds with *N*-alkyl/aryl hydroxylamines were one of the first methodologies implemented for nitrones syntheses and are still one of the most commonly applied nowadays. Oximes are also capable of nucleophilic substitutions with compounds with good leaving groups (Scheme 5).

**Scheme 6:**
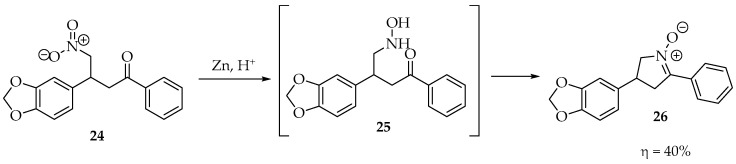
Example of an intramolecular cyclization between a ketone and a hydroxylamine formed in situ.

Starting with *N*-alkyl/aryl hydroxylamines or simply hydroxylamine, as shown in Scheme 5, countless examples exist in the literature for their condensation with aldehydes and ketones. The hydroxylamine moiety can be prepared in situ from nitro compounds, the most sensitive compounds, to avoid degradation in air. As an example, the 3-(benzo[*d*][1,3]dioxol-5-yl)-4-nitro-1-phenylbutan-1-one **24** was reduced by zinc dust in acidic media, affording the terminal hydroxylamine **25**, which readily condensed, intramolecularly, with the carbonyl to produce the cyclic 3,4-dihydro-2*H*-pyrrole-1-oxide derivative **26** (Scheme 6) [11].

**Scheme 7:**
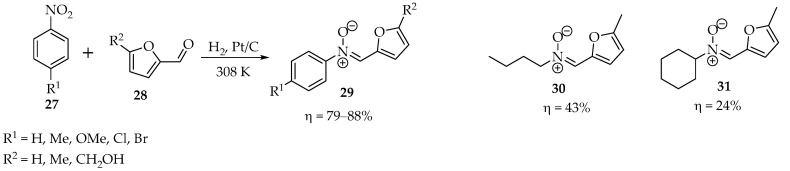
Example of a one-pot transformation of nitro compounds into nitrones.

In the hope of finding a more sustainable methodology, Cisneros et al. tried a different, one-pot approach, using nanocatalysts for the selective hydrogenation of nitro groups, followed by the formation of hydroxylamines and cyclization into nitrones (Scheme 7) [31]. Excellent results were achieved with 0.2 wt% Pt/C at 308 K, using 5 bar hydrogen pressure as a reagent, even in the presence of alkenes and alkynes. Unfortunately, this system only shows selectivity in the case of nitroaryl compounds, having poor yields in the case of nitroalkyl compounds, such as in the cases of nitrones **30** and **31** (Scheme 7) [31].

The direct reaction between hydroxylamines and aldehydes using ball-milling was performed without solvent, with better results than microwave irradiation [32]. The frequencies were optimized to 30 Hz and hydrogen carbonate was used as a base to increase hydroxylamines’ nucleophilicity, because commercial hydroxylamines are acidified with hydrogen chloride to avoid evaporation [32]. From the three hydroxylamines used, the *N*-methylhydroxylamine needed to be recrystallized twice with AcOEt/EtOH at −20 °C prior to usage, which depicts the low stability of hydroxylamines. In the conventional methodology, the MgSO_4_ can be used as a desiccant, and the reaction is performed at room temperature in dichloromethane for some hours [33].

**Scheme 8:**
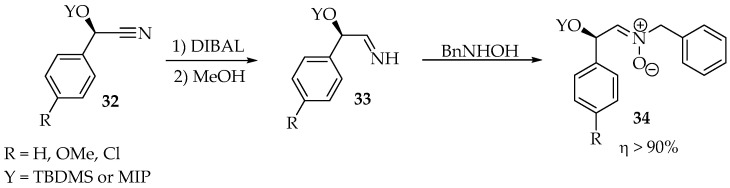
Cyanohydrins’ **32** reduction to imines **33**, followed by transamination with *N*-benzylhydroxylamine to produce *N*-benzylnitrones **34**.

The scope of this reaction has been extended to imines, nitriles, and cyanohydrins—in the latter case, they must be converted to imines first (Scheme 8) [34]. That reduction is performed by diisobutylaluminium hydride (DIBAL) at −10 °C, followed by protonation with methanol. Finally, after adding a hydroxylamine derivative, the transamination takes about 4 h at room temperature.

Nucleophilic substitutions with oximes are less frequent, the main reason being the lower reaction yields. This functional group presents ambidexterity, attacking electrophilic species with nitrogen or oxygen atoms. Furthermore, it forms diastereoisomeric products [35].

**Scheme 9:**
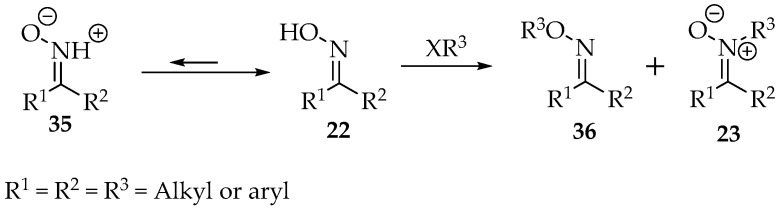
Oxime alkylation produces a mixture of oxime *O*-ether **36** and nitrone **23**.

The oxime structure **22** is a tautomeric equilibrium with the nitrone form **35**, though the first is thermodynamically more stable (Scheme 9) [36]. As nitrogen is considerably more acidic in oximes than in hydroxylamines, the strength of the nucleophilic attack by this atom (nitrogen) should be inferior. Increasing the mixture’s pH will further improve the nucleophilicity of the oxygen [2]. In relation to the oxime, the *E*-oxime also favors substitution on the oxygen, when compared with the *Z*-conformer, mainly due to steric reasons [37]. The same reasoning explains the formation of oxime *O*-ether when ketoximes are used instead of aldoximes [36].

Furthermore, in the case of aryl oximes, the presence of electron-releasing groups yields more ether **36**, whereas the electron-withdrawing groups produce more nitrone **23** (Scheme 9) [4]. Finally, the electrophile’s nature has another impact on the product ratio. Using iodide reagents makes the reaction faster and improves the nitrones’ yield. The bromine and chlorine substituents prefer the formation of ethers’ [35]. Fortunately, their separation seems easy, as they recrystallize the nitrone from an ether–alkane solvent mixture [37].

**Scheme 10:**
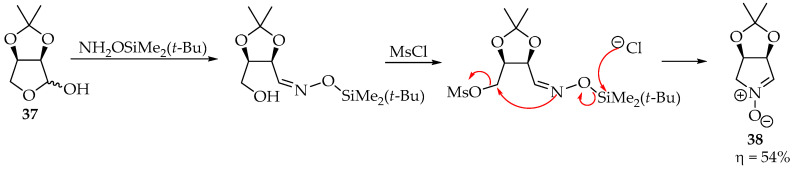
Example of a one-pot nitrone synthesis using NH_2_OSiMe_2_(*t*-Bu).

A particular system in which nitrones are obtained in good yields is the case of activated hydroxyl substituents (as leaving groups from the electrophile) (Scheme 10). This has been achieved by mesylating aldose oximes with methanesulfonyl chloride (MsCl) in pyridine [38]. An alternative is using Mitsunobu conditions: adding triphenylphosphine and diisopropyl azodicarboxylate reagents to the alcohol, to activate the hydroxyl group [35].

Although it is not a true nucleophilic substitution, nitrones have also been produced from oximes by Chan–Lam coupling with vinyl and aryl boronic acids [39]. The reaction showed good yields using copper(II) acetate as a catalyst when pyridine and 1,2-dichloroethane were used as solvents.

Finally, no interconversion between the oxime *O*-ether and the nitrone was observed when the reaction was left for a longer reaction time, suggesting good stability of both products when formed [35]. However, there is a special case in which it was achieved with a copper(I) catalyst [40].

### 2.3. Additions to Multiple Bonds

**Scheme 11:**
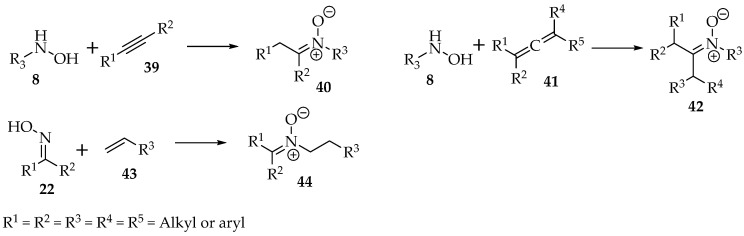
General examples of addition reactions of hydroxylamines **8** and oximes **22**.

Besides substitutions, the nucleophilic hydroxylamines **8** and oximes **22** can be added to alkynes **39**, allenes **41**, or alkenes **43** to produce nitrone derivatives (Scheme 11).

**Scheme 12:**
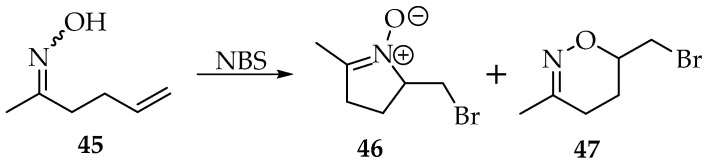
Example of Grigg’s intramolecular synthesis.

The first reaction using oximes addition to alkenes became famous with the studies of Grigg, hence receiving his name [3,41]. However, in Grigg’s synthesis, many side reactions may occur, like in the nucleophilic substitutions. The ambidexterity of oximes results in nitrones and oxime *O*-ethers, depending on the reaction conditions and the nature of the reagents. Furthermore, both the obtained nitrone derivatives can react with alkenes through 1,3-dipolar cycloadditions, producing heterocyclic compounds. Many examples showing this reactivity of nitrones can be found in the literature [42]. The examples provided by Grigg’s synthesis show that it is difficult to obtain the isolated nitrone. For instance, oxime **45** undergoes an intramolecular cyclisation to produce nitrone **46** in low yield and oxazine **47** in good yield (61%) (Scheme 12) [43].

**Scheme 13:**
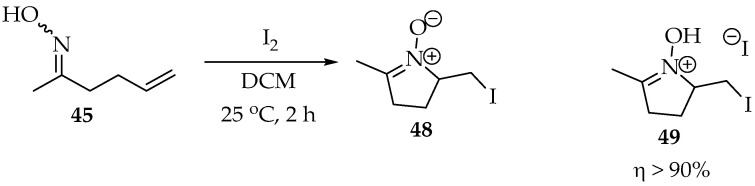
Intramolecular Grigg’s synthesis, using iodine as an electrophile to activate the double bond.

From Grigg’s studies, it became clear that the alkene should contain an electron-withdrawing group or be activated by an electrophile or a Lewis acid [41,43]. For example, in the fortunate experiment represented in Scheme 13, iodine was used as the electrophile, and the nitrone **48** was obtained as the sole product, although isolated in its salt form. This was lucky because, as explained in the previous section, the iodine tends to form nitrone products, and the simple oxime does not cause substantial steric hindrance. In a more recent study, it was calculated that the transition state’s energy could be halved when bases are added, such as potassium *tert*-butoxide [44]. The base’s cation has a high impact on stabilizing the transition state’s negative charges.

**Scheme 14:**
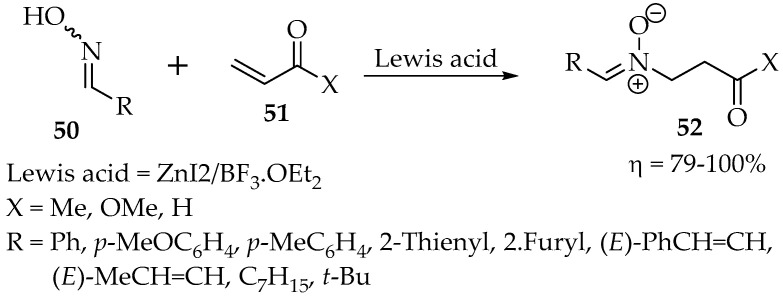
Nitrones synthesis through the reaction of oximes with α,β-unsaturated carbonyl compounds.

Finding suitable Lewis’s acids for this reaction can be arduous. In another study, in which several aldoximes were reacted with α,β-unsaturated carbonyl compounds, the best combination of Lewis acids was the proportion of 1:1 ZnI_2_/BF_3_•OEt_2_ (Scheme 14) [45]. When only zinc iodide was tried, a mixture with the oxime *O*-ether was formed, whereas the reaction was not completed with only the second component. According to Roca-López et al. [36], the nitrone is formed when there is an orthogonal disposition of the oxime with the alkene moiety.

One final discovery was presented in the reaction of ketoximes with cyclopropenes, which had high enantioselectivities and no ring-opening side products. A CuCl Lewis acid with different bulky ligands was used at 5 mol%, along with sodium *tert*-butoxide (30 mol%) under an inert atmosphere [46].

**Scheme 15:**
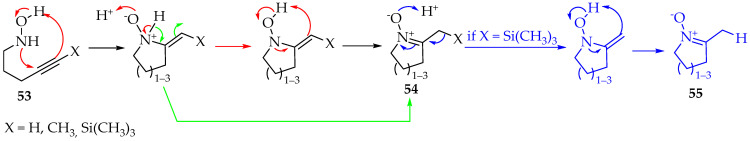
Intramolecular reaction between hydroxylamines and alkynes producing five to seven-membered cyclic nitrones.

Depending on the reagents used, the addition mechanism can be either a nucleophilic attack or radical formation. The second case is rarer, however, successfully achieved for the first time by Han et al. [47]. The radical initiator was diethyl diazenedicarboxylate, and 5-*exo*-trig selectivity was evident. Additions through both nitrogen and oxygen are possible, with the product favored by the previous selectivity. For the intramolecular reaction of hydroxylamine derivatives with alkynes, cyclic nitrones **53** are produced, and their ring members are between five and seven (Scheme 15). In the case where the hydroxylamine derivatives with alkynes are silylated, that is, X = Si(CH_3_)_3_, the substituent is lost and nitrones **55** (Scheme 15) [48] are obtained. Between the three substituents presented in Scheme 15, the methyl group has the worst results for not polarizing the triple bond. Therefore, a slower reaction and worse yields are expected. This feature is generally common in Cope eliminations [49].

Mechanistically, this synthesis is concerted in the first step, with no radicals being formed (Scheme 15) [48,49,50]. Then, the hydron is transferred from nitrogen to oxygen in a bimolecular process, requiring another molecule with labile hydrons, which can be obtained from the solvent (Scheme 15—red step). Beauchemin et al. [50] have calculated the energy involved, and found this to be more favorable than direct intramolecular hydron transfer between the two atoms. Finally, the last step towards forming the nitrone **54** is similar to the first step, with a concerted mechanism. Fox et al. [48] proposed that the hydron is transferred directly from nitrogen to the α carbon, due to tautomerization (Scheme 15—green step). Regardless of the mechanism, the reaction was tried successfully between a hydroxylamine derivative and an alkyne from two distinct molecules. The solvent used was propanol, at 110 °C for 14 h. The yield was good (52%) [50]. Excellent yields were obtained in another study for intramolecular reactions, using 5 mol% AgOTf in 1,2-dichloroethane, at room temperature, to a maximum of 4 h [51].

**Scheme 16:**
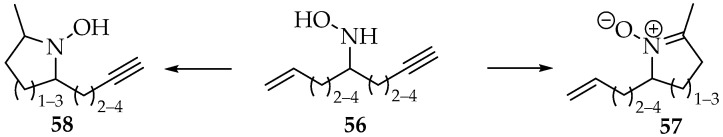
Competitive intramolecular reactions of hydroxylamines with alkenes and alkynes.

A comparison in reactivity of hydroxylamines with alkenes or alkynes has also been highlighted, using the hydroxylamine **56**, which contains the three moieties (Scheme 16). The preferred product of the hydroxylamine **56** containing the three moieties depends on the ring size and the alkene/alkyne substituents [48,49].

As a rule, the nitrone **57** is formed when the product’s ring is six-membered. If not, the hydroxylamines **58** are preferred. However, as mentioned previously, the presence of alkyl substituents increases the activation energy. Hence, when the alkene is substituted and the alkyne is terminal (or silylated), the nitrone is always favored over the hydroxylamine [48,49].

**Scheme 17:**
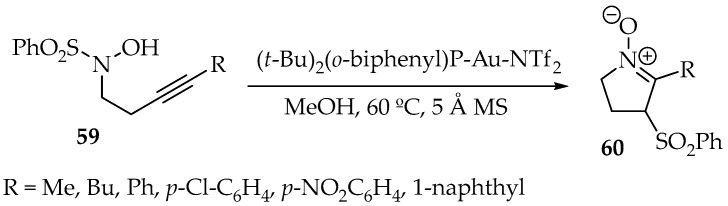
Intramolecular reaction between a hydroxylamine and an alkyne, with concomitant 1,3-tranfer of a sulfonyl substituent.

In a new gold catalysis reaction, the intramolecular cyclization of the hydroxylamine **59** with its alkyne moiety, with a concomitant transfer of a sulfonyl group from *N*- to α-position, has been achieved (Scheme 17). In addition to the Au catalyst, methanol at 60 °C and molecular sieves were used. The resulting nitrone **60** was obtained with yield ranging between 39% and 88% [52].

**Scheme 18:**
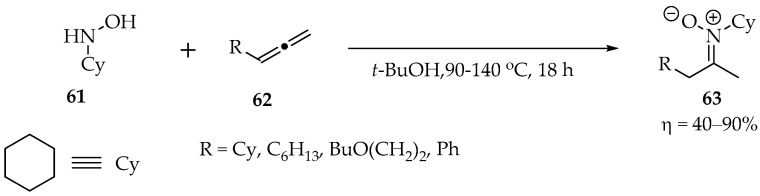
Example of a hydroxylamine reaction with allenes.

In another study by Beauchemin et al., the hydroxylamines were also reacted with allenes. The temperatures ranged between 90 and 140 °C in *tert*-butanol for 18 h. The yields were very good in most cases (Scheme 18). Mechanistically, the reaction is similar to the one with alkynes [53].

### 2.4. From Nitroso Reagents

**Scheme 19:**
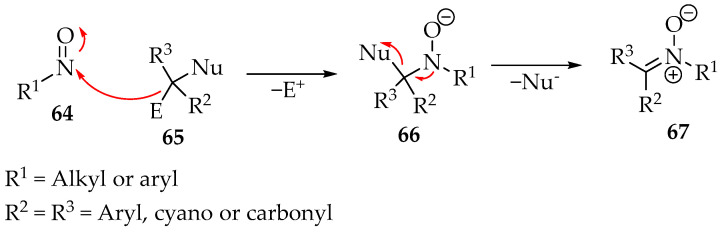
Proposed mechanism for the reaction between nitroso compounds **64** and active methylene species **65** to produce nitrones **67**.

Nitrones can be prepared from nitroso compounds **64** (Scheme 19) by reacting with structures resembling methylene **65**. For example, the diazo compounds fulfill that role. In essence, the reactive carbon must be substituted by a group that can leave with the bond electrons (nucleophile Nu^−^) and another group leaving positively charged (electrophile E^+^). In the case of the azo compounds, that substituent executes both functions. A possible mechanism is represented in Scheme 19.

The nitroso compound **64** is in a high-oxidation state, so acting primarily as an electrophile is natural. As a result, a hydroxylamine’s conjugated base forms **66** as an intermediate. Then, the nucleophile is expelled from the molecule by the nitrogen’s electron pair (Scheme 19). This theory has already been supported in forming imines when the nucleophile group is absent by eliminating a water molecule from the intermediate **66** [3,54]. Nevertheless, more conclusive studies are required.

**Figure 4:**
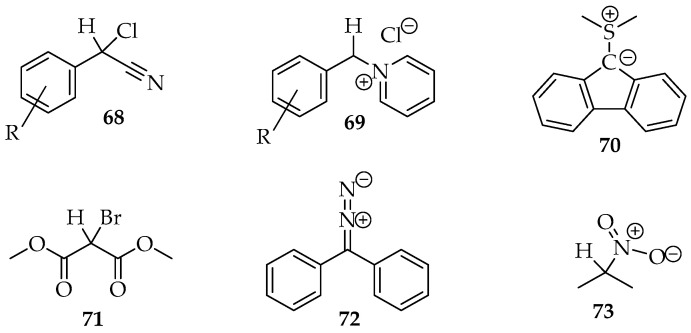
Some examples of active methylene compounds.

Many examples of this reaction type were reviewed by Hamer and Macaluso [4]. The construction of species **65** usually consists of aryl, cyano or carbonyl substituents in positions R^2^ and R^3^ (Scheme 19). Hence, the hydron or another electrophile is eliminated more easily from the molecule, for example, by adding a base. Several compounds with halogens, diazo, or nitro groups (Figure 4) have been reported for the nucleophile. The nitro compounds with α-hydrogens **73** are excellent; the nitro substituent facilitates hydron removal through structure stabilization by resonance and acting as the nucleophile leaving group [3]. Additionally, pyridinium salts **69** and sulfur ylides **70** (Figure 4) are also very good nucleophiles [3,4].

**Scheme 20:**
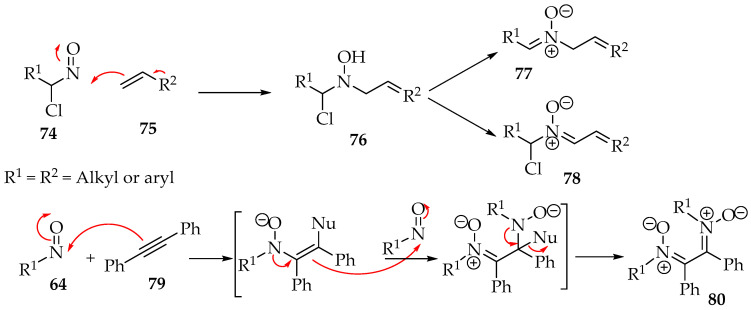
Electrophilic addition of nitroso compounds to alkenes or alkynes.

The nitroso reagents can also react with multiple bonds, which has led to the discovery of new nitrone syntheses in the last two decades. Instead of having an electrophile in the structure, the π bond provides the electrons to the nitroso group. Then, there is either a nucleophile, as in compound **75**, or another π bond, as in alkyne **71**, to set up the final nitrone (Scheme 20).

**Scheme 21:**
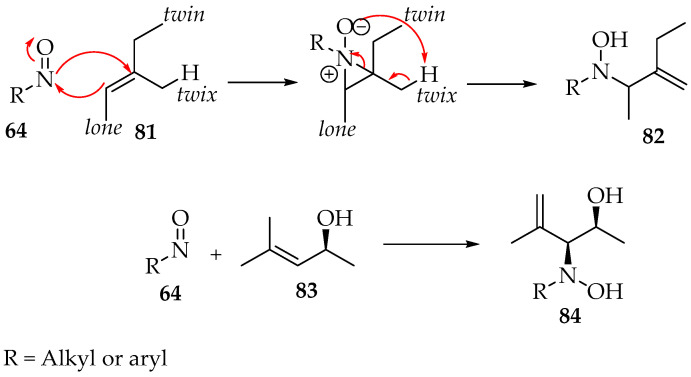
Regio- and stereoselectivity of the addition of nitroso compounds to alkenes.

The preparation of a compound like **77** requires, for example, a geminal chloronitroso reagent, which has been synthesized by Bou-Moreno et al. [55] from secondary nitro substituents and oxalyl chloride. Then, Adam and Krebs explored how it reacts with the alkene [56]. First, an aziridine *N*-oxide is believed to be formed as an intermediate (Scheme 21). Then, in the transformation to the hydroxylamine derivative **82**, the hydron captured by its oxygen belongs to the *twix* position (Scheme 21). Moreover, in the case of the formation of chiral derivatives, the carbon-nitrogen bond formation can be directed by adjacent hydroxyl groups to the *threo*-stereochemistry, as exemplified by compound **84** (Scheme 21) [56].

**Scheme 22:**
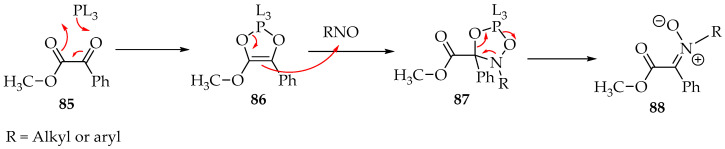
Possible mechanism for the reaction of nitroso compounds with 1,2-dicarbonyls, in the presence of a phosphine derivative.

One more excellent reaction is the formation of nitrones from 1,2-dicarbonyl compounds in the presence of *N*,*N*,*N*′,*N*′,*N*″,*N*″-hexamethylphosphanetriamine [P(NMe_2_)_3_ = PL_3_]. The phosphine derivative transforms the 1,2-dicarbonyl **85** into a dioxaphosphole derivative **86**, which then reacts with the nitroso compound (Scheme 22) [57]. The mechanism has still not been verified experimentally. Still, the most accepted possibility is the formation of dioxazaphospholidine derivative **87**, which, upon ring opening and elimination of the phosphine moiety, gives the desired nitrone **88** (Scheme 22) [57].

**Scheme 23:**
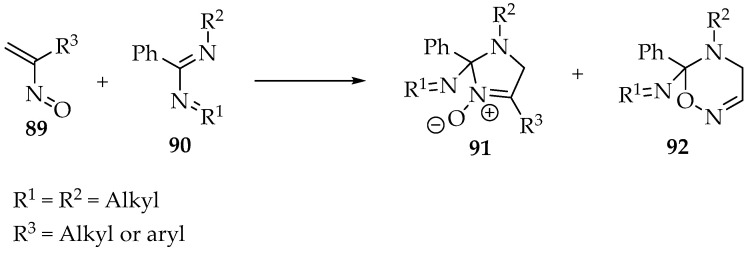
Reaction of an α,β-unsaturated nitroso **89** with an imidamide **90**.

The last illustrative reaction with alkenes combines an α,β-unsaturated nitroso **89** with an imidamide **90** (Scheme 23) [3]. It is an ingenious protocol to produce imidazoline oxides **91**, and many other cyclic nitrones can be obtained. However, one must be aware that nucleophilic attack by the oxygen is now possible, leading to the formation of 5,6 dihydro-4*H*-1,2,5-oxadiazines **92** as by-products [3].

**Scheme 24:**
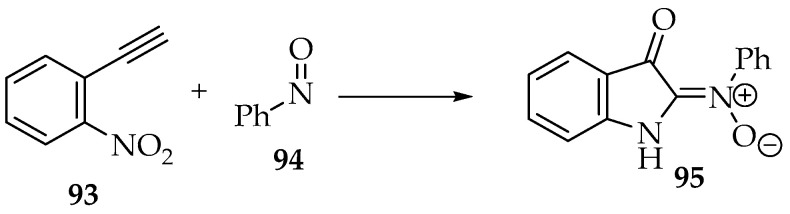
Reaction between 1-ethynyl-2-nitrobenzene **93** and nitrosobenzene **94,** producing nitrone **95**.

The reactions between nitroso compounds and alkynes are not explored well, at least to produce nitrones. The most common product is the previously presented dinitrone **80** (Scheme 20) [4]. Using a gold catalyst, one of the nitrone moieties is reduced to an imine [52]. Hamer and Macaluso have shown an unexpected product [4]. The reaction between 1-ethynyl-2-nitrobenzene **93** and nitrosobenzene **94** forms a nitrone with an indoline heterocycle **95** (Scheme 24) [4]. This results from reducing the nitro group to an amine and one nitrone moiety to an imine (Scheme 16), followed by an intramolecular cyclization.

**Scheme 25:**
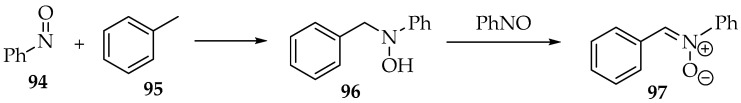
Reaction of nitrosobenzene with toluene, with a final oxidation step to nitrone.

One last methodology through which nitroso compounds may form nitrones takes advantage of its reduction potential. For example, toluene and cycloheptatrienes react with one molecule of nitrosobenzene **94**, furnishing hydroxylamines **97** (Scheme 25). Then, the hydrogen atom of the hydroxyl group transfers to another nitrosobenzene **94** molecule, oxidizing the hydroxylamine **97** to a nitrone **98** (Scheme 25) [4,58].

### 2.5. From Nitro Compounds

**Scheme 26:**
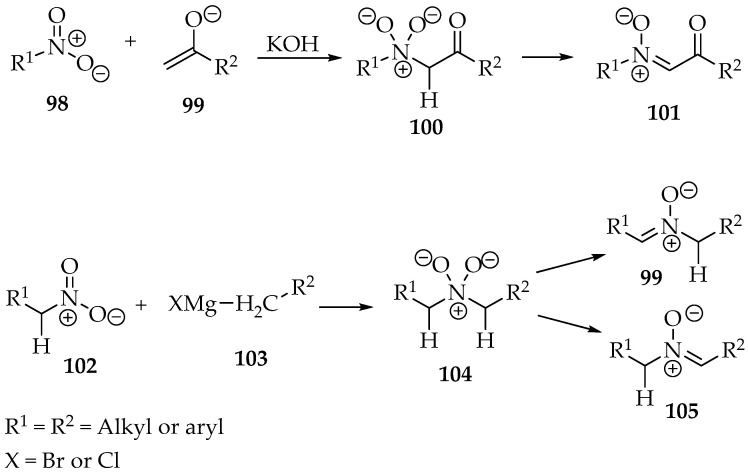
Synthesis of nitrones through condensation reaction with nitro compounds.

Nitrones can be prepared by the alkylation of nitro groups. The nucleophilic carbons reported are usually from enolates **99** or Grignard reagents **103** (Scheme 26). Then, either the nitro compound already has an α-hydron **102** or the newly attached carbon must have it to form a double bond after condensation (Scheme 26) through water elimination.

**Scheme 27:**
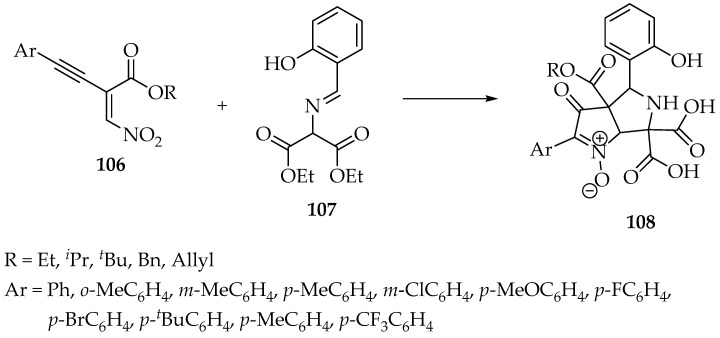
Synthesis of bicyclic nitrone derivatives **108** from a nitro enyne **106** and a 2-(benzylideneamino)malonate **107**.

Nowadays, these reactions are rarely used; however, a more recent work aiming to study organocatalytic reactions on electron-deficient alkynes developed a new synthesis of bicyclic nitrones **108** using ethyl 2-(nitromethylene)-4-arylbut-3-ynoate **106** and diethyl 2-[(2-hydroxyben-zylidene)amino]malonates **107** (Scheme 27) [59]. The proposed mechanism is quite complex, but the authors could identify several intermediates by high-resolution mass spectrometry. Additionally, they suggest that the reaction starts with a conjugate addition of the deprotonated 2-[(2-hydroxyben-zylidene)amino]malonate to the enynoate **107** (Scheme 27) [59].

### 2.6. From N-Hydrxyamide Compounds

The preparation of nitrones from *N*-hydroxamides, also known as hydroxamic acids, is not a frequently used method. Two interesting examples deserve to be highlighted, both employing a similar strategy: the protection of oxygens, followed by reduction and deprotection.

**Scheme 28:**
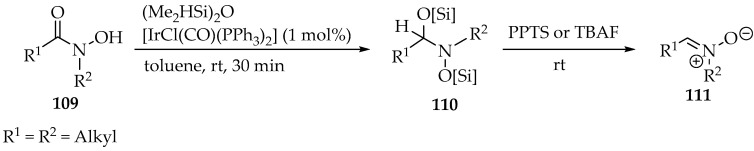
Partial reduction in *O*-protected *N*-hydroxyamides **109** with a siloxane producing nitrones **111**.

In the first example, the 1,1,3,3-tetramethyldisiloxane [(Me_2_HSi)_2_O] was used as protecting group and reductor, concomitantly (Scheme 28) [60]. Nevertheless, the iridium catalyst was essential for obtaining the nitrone derivative **111**, and the method was applied to a few substituents in the hydroxamic acid **109**, demonstrating high chemoselectivity due to the bulky oxygen-protected atoms (Scheme 28, structure **110**). Finally, the deprotection with PPTS or TBAF also promotes the elimination into nitrone **111** (Scheme 28).

**Scheme 29:**
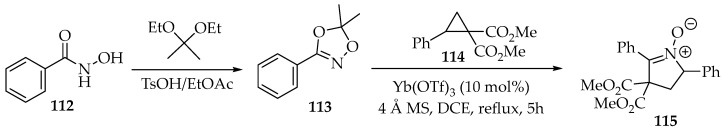
Partial reduction in *O*-protected *N*-hydroxyamide **113** through a [3+2]-annulation with a cyclopropane, producing nitrone **115**.

In the second example, protection was achieved through cyclisation with acetals, resulting in dioxazole **113** (Scheme 29) [61]. Then, the reduction was disguised as a [3+2]-annulation with dimethyl 2-phenylcyclopropane-1,1-dicarboxylate **114** (Scheme 29) [62]. This was performed in the presence of molecular sieves (MSs) and an ytterbium Lewis acid, with the nitrone **115** being produced and purified by silica gel chromatography [62].

### 2.7. Oximes Rearrangement

**Scheme 30:**
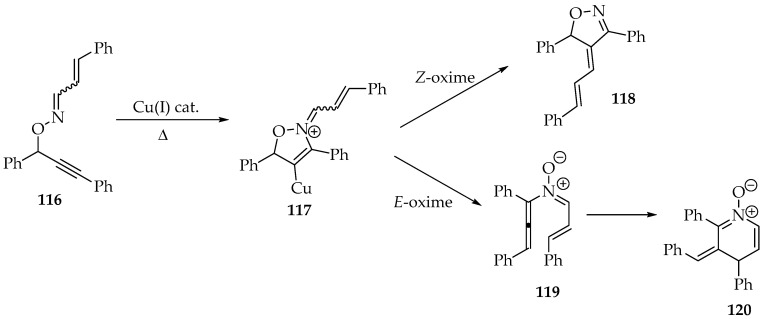
The *O*-propargyl oxime **116** transformation into cyclic nitrones **120**, through a copper(I) halide catalysis.

Although this rearrangement is rare, due to the oximes’ reported stability, it was achieved by Nakamura et al. [40]. Using a copper(I) halide catalyst, the authors successfully converted *O*-propargyl oximes 116 into stable cyclic nitrones **120** (Scheme 30) [40]. Only the *E*-oxime **119** could be transformed in this way, as the other diastereomer would undergo an alkylidene transfer, forming the oxazoline **118**, which keeps the oxazoline ring from the intermediate **117** (Scheme 30).

Mechanistically, it was proposed that the copper catalyst would facilitate the 5-*endo*-dig cyclization, which would then be removed for another cycle. As a result, the *N*-allenyl imine oxide **119** is produced, which undergoes further rearrangement to the dihydropyridine oxide derivative **120** (Scheme 30).

## 3. Nitrones Transformations

Nitrone transformations are a significant topic because they allow for the synthesis of new compounds that can become critical bioactive compounds. However, an exhaustive description of the existing nitrones transformations will not be provided herein, as it is not the objective, the review size is limited, and, above all, because an excellent review was published in 2019, which presents and discusses the main transformations [9]. Nevertheless, a few vital transformations and/or more recent ones will be addressed.

### 3.1. Beckmann Rearrangement

Nitrones rearrangements are often related to the N-O bond liability, and they are quite useful for the preparation of lactams. The Beckmann rearrangement is an exceptional example; however, prolonged irradiation times favor amide formation [63].

**Scheme 31:**
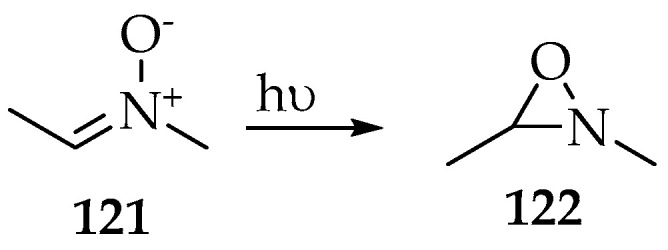
The isomerization of nitrone **121** to oxaziridine **122** after excitation to the S_1_ state by irradiation (λ 313 nm).

The Beckmann rearrangement corresponds to the oxygen shift from the nitrogen to the nitrone’s carbon, so it can be seen as a subsequent transformation of oxaziridines, which were found as intermediaries in the production of amides and lactams [64]. Actually, one of the most important isomerization transformation of nitrones is its cyclization into a three-membered ring by those same three atoms, named oxaziridine. This transformation is usually directly related to the degradation of nitrone under poor storage conditions; however, it can also be induced photochemically. For example, it was found that the conversion into 2,3-dimethyl-1,2-oxaziridine **122** (Scheme 31) could be achieved upon irradiation of (*Z*)-*N*-methylethanimine oxide **121** with a mercury lamp whose wavelength is 313 nm [65,66]. The singlet electronic ground state’s electron S_0_ is excited to the first singlet excited state S_1_, and then the oxygen forms a single bond with the carbon.

**Scheme 32:**
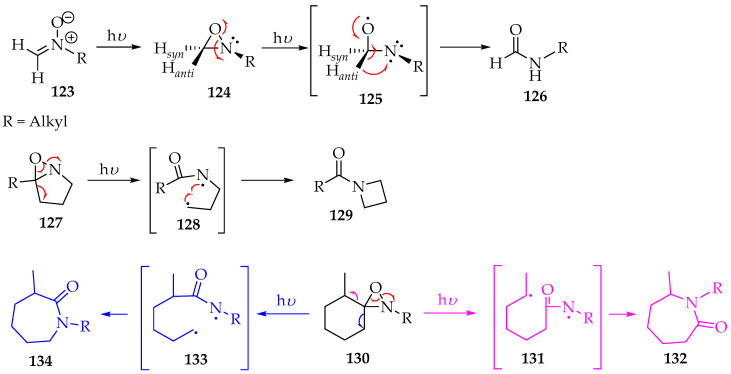
Nitrones photoisomerization, via oxaziridine intermediates.

Indeed, the Beckmann rearrangement is a versatile method for transforming nitrones into other valuable organic compounds. For instance, irradiating the nitrone derivatives **123** at λ 313 nm will convert them into oxaziridine derivatives **124** (Scheme 32) [64]. After isomerization into oxaziridine, radiation also promotes homolytic N-O bond cleavage, producing a transient diradical structure **125**, which, upon the *anti*-hydrogen shifting to the nitrogen, gives the final amide derivative **126** (Scheme 28) [64]. Accordingly, the authors believe that the dissociation of the C-H activation energy is very low, which explains the good selectivity. However, for bicyclic oxaziridine derivatives **127**, both cycles are broken to form the biradical intermediate **128**, which subsequently undergoes an intramolecular cyclisation to amide derivatives **129** (Scheme 32). In another example, with spirooxaziridine derivatives **130**, a mixture of the two lactams **132** and **134** was obtained, although in a highly selective manner towards lactam **134**, in a ratio of 95:5 (Scheme 32). The stereochemistry is governed by stereo hindrance rather than the lower energy state, so the reaction proceeds via the less stable radical **133** rather than through the more stable one, **131** [64].

**Scheme 33:**
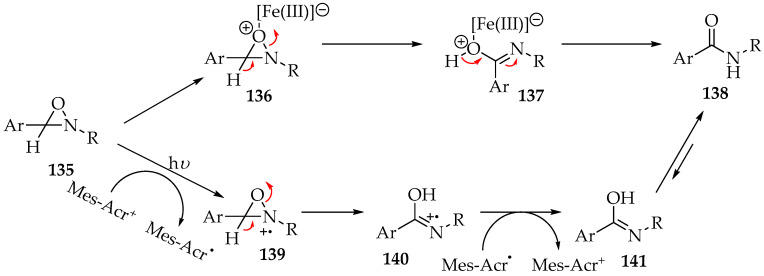
The transformation of oxaziridine **135** into amide **138**, through the iron(III) complex, and by photoredox activation with 9-mesityl-10-methylacrydinium tetrafluoroborate (Mes Acr^+^).

Other strategies have been implemented for the second step of the reaction (transformation of the oxaziridine **135** into the amide), namely the addition of bases and Lewis acids (Scheme 33) [63,67,68]. In the case of acids such as iron(III) sulfate, the mechanism is purely ionic, as shown in Scheme 33 [63]. First, there is the formation of the iron complex **136**, followed by the oxaziridine ring opening and the decomplexation into the amide **138**. For the bases, it is advised to use photocatalysis in combination with a weak base [68]. The reason is that the hydron shift is the limiting step, and it is weakly acidic [68]. Strong base usage is possible, for example, with LDA or LTMP, but it also leads to the formation of by-products [67]. However, the addition of a photocatalyst, such as 9-mesityl-10-methylacrydinium tetrafluoroborate (Mes-Acr^+^), will oxidize the oxaziridine **135**, making the hydron more acidic and reactive with weak bases.

### 3.2. Dipolar Additions

**Figure 5:**
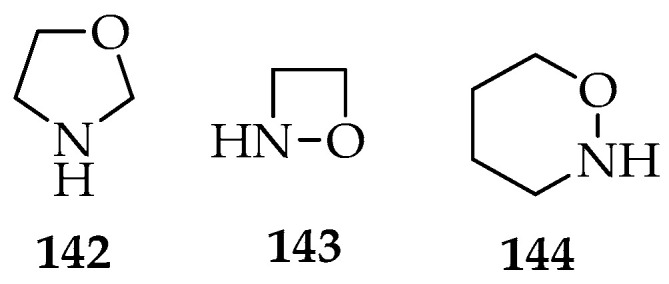
Oxazolidine **142**, 1,2-oxazetidine **143,** and 1,2-oxazinane **144** structures.

The dipolar additions to nitrones are a versatile method for obtaining new heterocyclic compounds. The most frequent system of dipolar additions is the [3+2]-cycloaddition, which produces oxazolidine analogous **142**. The [3+1]-cycloadditions form 1,2-oxazetidine **143** type compounds, whereas the [3+3]-cycloadditions form 1,2-oxazinane **144** derivatives (Figure 5). Sometimes, other heteroatoms are incorporated into the rings, depending on the initial substrates, and intramolecular cycloadditions also occur, resulting in the formation of two new rings.

Generally, dipolar addition involves the nitrone’s oxygen addition to one of the atoms involved in a π bond, and the other atom from the same π bond or another atom in a conjugated π system will form a new bond with the nitrone α-carbon and compensate the positively charged nitrogen. They are usually considered concerted mechanisms [69,70].

#### 3.2.1. [3+1]-Cycloadditions

**Scheme 34:**
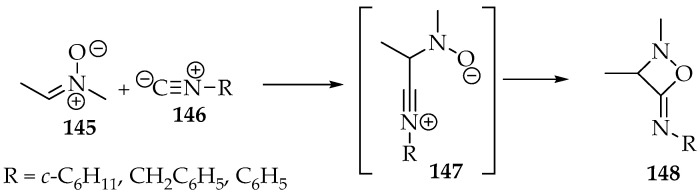
The [3+1]-cycloaddition of nitrone **145** to isonitriles **146**, producing 1,2 oxazetidin-4-imine **147**.

This reaction appears to be restricted to the addition of isonitriles **146** in the presence of BF_3_•OEt_2_ and triethylamine [71]. It is completed in two minutes at −40 °C, producing 1,2-oxazetidin-4-imines **147** (Scheme 34). Not many examples were reported, and the yield is dependent on the isonitrile substituent; good yields (>50%) were obtained with aliphatic carbons attached to the isonitrile **146**, whereas with aromatic carbons, the yield was lower (η = 25%).

#### 3.2.2. [3+3]-Cycloadditions

**Scheme 35:**
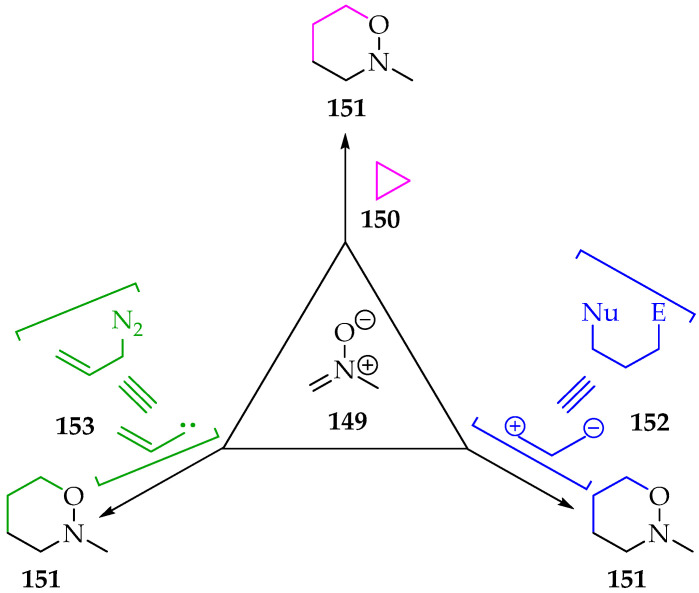
The possibilities for the [3+3]-cycloaddition of nitrone type **149**.

As mentioned before, the [3+3]-cycloaddition type often forms 1,2-oxazinane products **144** (Figure 4), although, depending on the reagents used, their final structure can present substituents, including *N*-alkyl groups (Scheme 35). The [3+3]-cycloadditions are, in general, less common [72], and their application using nitrones is also scarcer [73]. Among the ways to perform this reaction is the reaction of nitrones **149** with a three-membered ring derivative **150** (Scheme 35). By breaking one σ bond, the ring’s steric stress is released, and a three-atom bridge is added between the nitrone’s carbon and oxygen atoms. Nitrones’ reaction with cyclopropanes is relatively recent, with the debut dating back to the beginning of the twenty-first century, in a work of Young and Kerr [74]. Soon, it was found that the reaction followed a specific asymmetry, and a stepwise mechanism was suggested [75,76]. The nitrone’s oxygen first attacks one of the cyclopropane derivative’s more electrophilic carbons, forming a C-O bond and breaking the cyclopropane σ bond. This first step is the rate-limiting one and yields a high-energy zwitterionic intermediate with a negative charge on the other carbon atom of the broken σ bond and the positive charge on the nitrogen. Naturally, groups that stabilize the carbanion formed will contribute to lowering zwitterionic intermediate energy. The last step is the formation of the C-C bond to yield the 1,2-oxazinanes **151** [75,76].

**Scheme 36:**
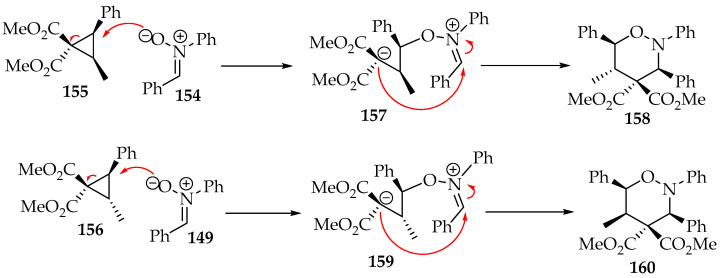
The mechanistic proposal for the [3+3]-cycloaddition of nitrones to diastereoisomeric cyclopropanes *cis*-**155** and *trans*-**156**.

The cyclopropane’s substituents and stereochemistry dictate the regioselectivity of the 1,2-oxazinane. A detailed example is the reaction of the nitrone **154** with each cyclopropane diastereoisomer **155** and **156** (Scheme 36) [76]. The orientation of the oxygen attack and the location of the new σ bond are anti to the broken σ bond, a feature also observed in other examples [77,78]. As a result, the stereochemistry of the cyclopropane carbon not involved in the broken σ bond is inverted, and the *cis*-2-methyl-3-phenylcyclopropane **155** leads to a 5,6-*trans*-1,2-oxazine **158**, whereas the *trans*-2-methyl-3-phenylcyclopropane **156** forms the 5,6-*cis*-product **160** (Scheme 36). This reaction has only been tested with *Z*-nitrones, and the favored products that present the 3,6-*cis*-diastereoselectivity are depicted in Scheme 36 [76].

So far, no theoretical calculation has been performed on this system, and the rational mechanism depicted in Scheme 36 is based on the transition state proposed in Karadeolian and Kerr’s study [76]. Fundamentally, the carbanion binds to the nitrone’s carbon from the opposite side of the bulkiest group (phenyl). Nevertheless, other examples also establish this reaction’s high stereoselectivity [79].

**Scheme 37:**
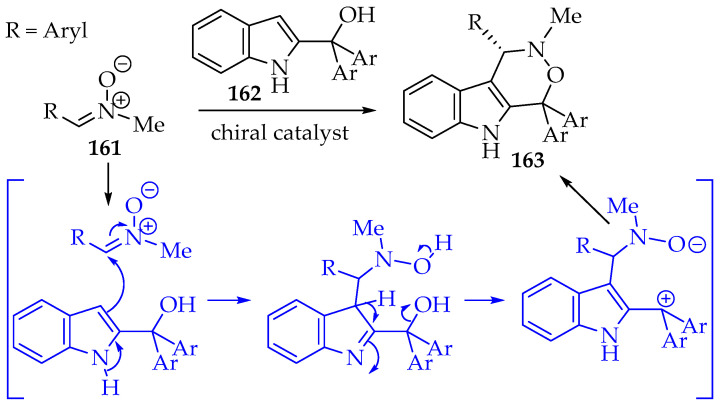
[3+3]-Cycloadditions between nitrones **161** and indole derivatives **162**.

The next [3+3] cycloaddition is the reaction of nitrones with open-chain dipoles prepared in situ, with an anionic/nucleophilic and a cationic/electrophilic part (Scheme 35, structure **152**). The carbanions may be produced at the α-carbon of carboxylic acids or from silylated compounds. Alternative systems are enamines and heteroatoms. On the positively charged part, activated hydroxyl groups, for example, acetoxy, and halogens are the main strategies applied [7,9,80]. An interesting example is the 2-(1-hydroxyalkyl)indole **162**, which has a nucleophilic character at the indole carbon C-3, and the 2-substituent acts as the electrophilic center (Scheme 37). The application of a chiral catalyst (phosphate derivative) resulted in good enantioselectivity of the tricyclic product **163**. Additionally, the authors proposed a mechanism that explains the stereoselectivity and also evaluates the reaction scope of both indole and nitrone derivatives [81].

**Scheme 38:**
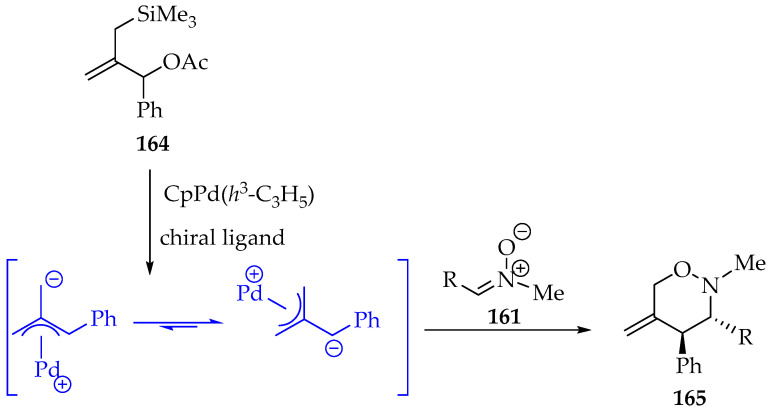
Another example of [3+3]-cycloadditions between nitrones **161** and open-chain dipolar compounds obtained in situ.

Another interesting example involves the use of silylated compounds, for instance, 1-phenyl-2-((trimethylsilyl)methyl)allyl acetate **164**, which, upon palladium catalysis, can form in situ an open-chain dipolar intermediate that reacts with nitrones, giving 1,2-oxazines **165** in high yield (Scheme 38) [82]. Moreover, the reaction scope was investigated, and a high stereoselectivity towards the *trans* isomer was observed.

**Scheme 39:**
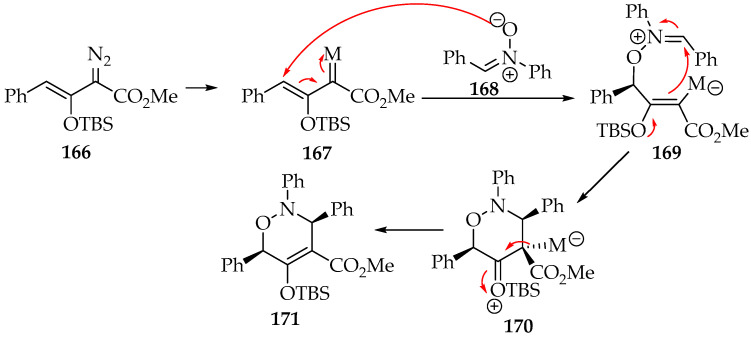
The [3+3]-cycloaddition of the nitrone **168** to the metallo-vinylcarbene **167**.

Regarding the vinyl carbene reagents, they have been prepared from vinyl diazo compounds **153** (Scheme 35) by coordinating it with a metal [7,9]. These metallo-vinylcarbenes of the Fischer type are generally electrophilic, due to the weak π-backdonation from the metal to the carbon. Considering the reaction of the vinyl diazo compound **166** as an example, there is in the first step the metal insertion, forming the metallo-vinylcarbene **167** (Scheme 39). Thus, as a result of the vinyl compound polarity inversion, the [3+3]-cycloaddition first goes through an addition of the nitrone’s oxygen at the β-unsaturated carbon, forming the intermediary **169**. Then, the cyclization is completed, not due to the nucleophilic character of the carbon bound to the metal by itself, but due to the resonance of an electron releasing group at the α-carbon. The [3+3]-cycloaddition follows the *syn*-configuration and the 3,6-dihydro-1,2-oxazine 166 product is formed (Scheme 39) [83,84].

**Scheme 40:**
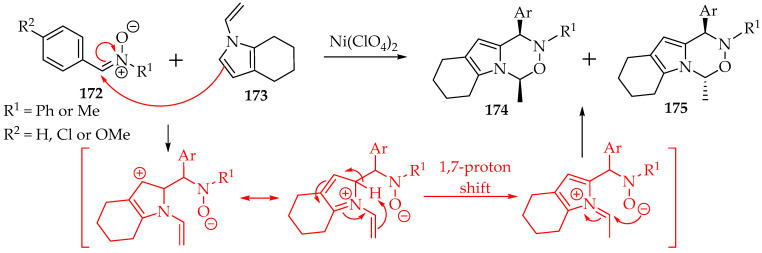
The reaction of the nitrone **172** with 1-vinyl-4,5,6,7-tetrahydro-1*H*-indole **173**.

The last chosen example is the reaction with the *N*-vinylindole derivatives **173** (Scheme 40). In this reaction, which requires a nickel(II) catalyst, to avoid a [3+2]-cycloaddition at the vinyl group, due to an interaction between the nitrone’s oxygen and the nickel ion [85], the mechanism is inverted, with the nitrone acting first as the electrophile and adding at the indole carbon C-2 (Scheme 36). It is followed by a 1,7-proton shift and the final cyclization to give the two possible isomers (Scheme 40). The obtained isomeric mixture showed a slight preference for the *cis*-diastereomer, though no chiral ligand was used [85].

#### 3.2.3. [3+2]-Cycloadditions

The [3+2]-cycloadditions involving nitrones have often been used to obtain new interesting compounds. This reaction is straightforward, yields high conversions, and can usually be performed in situ, immediately after the synthesis of the nitrone. It is also possible to carry out solid-supported reactions [86]. The reaction can be accelerated by increasing the pressure, raising the temperature, or using microwave irradiation. Additionally, a Lewis acid catalyst is frequently employed [3].

Nevertheless, this reaction type has two drawbacks: the variety of stereoisomers produced and its reversibility. Although protecting the nitrone might be helpful, the potential for changes in the stereoisomeric product ratio over time adds complexity. As a result, the products obtained under kinetic conditions may differ from those generated when the nitrone reacts for extended periods or at elevated temperatures (thermodynamic conditions). In such cases, one less favorable isomer may be transformed into a more stable one [87].

The most ubiquitous example is the reaction between a nitrone and an alkene, forming an isoxazolidine. The reaction can be either intramolecular or intermolecular. In the first case, bicyclic compounds are formed, and in the second case, the number of stereoisomers is larger (the alkene has more degrees of freedom).

**Scheme 41:**
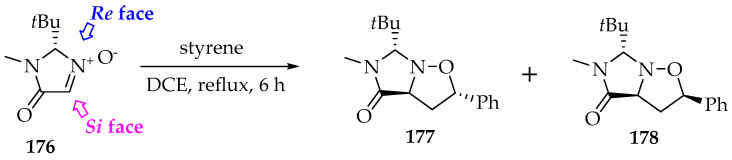
The [3+2]-cycloaddition of the nitrone **176** to styrene by the *Re* face, with the selective formation of the stereoisomers **177** (*exo*) and **178** (*endo*).

There are five roots for the asymmetry: the purity of the nitrone’s diastereomer *E/Z*, the localization of the addition on the *Re* or *Si* nitrone’s face (Scheme 41), the orientation of the alkene, the alkene’s diastereoisomerism, and the *endo/exo* attack. Fortunately, the products can be predicted with some accuracy, and there is often a preference for one or two products, obtained in good yields.

First, the reagents should be used as single stereoisomers. Then, the *Re*/*Si* face attack can be controlled by substituents on the nitrone or by appropriate chiral catalysts that crowd one face. In Scheme 41, the nitrone’s *Si* face **176** is hindered by the *tert*-butyl substituent (below the nitrone’s plane) [88]. In consequence, the styrene approaches through the *Re* face preferably (above the plane).

The alkenes’ substituents are the primary source of isomerism and are the most difficult to modify. The best is to understand their reaction models. Generally, the alkene’s orientation is such that its bulkier substituent is positioned at the *C*-2 of the isoxazolidine product (Scheme 41, products **177** and **178**). The electron-withdrawing groups, namely through the heteroatoms’ presence, also favor that position, because it is easier for the nitrone’s oxygen nucleophilic attack [3]. Thus, both steric and electronic factors drive the reaction asymmetry.

The main exception is the reaction with α,β-unsaturated carbonyls [89]. There, the conjugated system’s resonance leads to the formation of 4-carbonyl isoxazolidine derivatives. In the same way, the α,β-unsaturated nitriles, nitro alkenes, and vinyl sulfones result in the same substitution pattern (at *C*-3) [90,91,92].

In terms of *endo*/*exo* selectivity, the two possibilities are depicted in Scheme 41 for a cyclic nitrone. In the case of an *endo* preference, a *cis*-isoxazolidine is obtained, whereas the *exo* configuration produces the trans-isomer. The same happens on linear *E*-nitrones. For the *Z*-nitrones, the products’ diastereoselectivity is the opposite. The alkenes’ substituents also influence the *endo/exo* preference, though with less impact than for the other kinds of isomerism. Hence, mixtures of *trans*- and *cis*-isoxazolidines are often observed.

In general, the *exo* approach is followed to minimize steric energy. However, due to the dipolarity of some groups and hydrogen bonding, the *endo* configuration is sometimes observed. This is the case of the α,β-unsaturated nitriles and aldehydes [93]. For α,β-unsaturated ketones and esters, the increased bulkiness often results in the *exo* configuration [89].

**Scheme 42:**
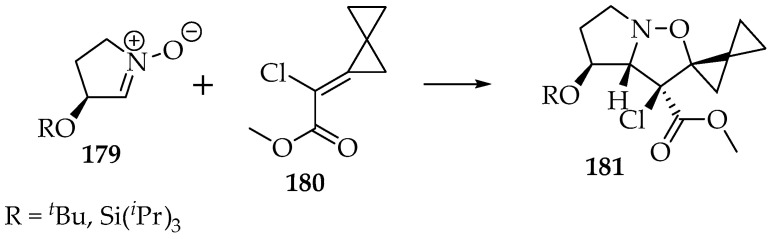
The [3+2]-cycloaddition of the nitrone **179** to the α,β-unsaturated ester **180** by the *Re* face, with the selective formation of the stereoisomer **181**.

That dipolarity is also helpful for discerning between two competing asymmetries (Scheme 42) [94,95]. In this example, both the ester and the alkyl chain prefer the *exo* configuration. Since the ester exhibits some dipolarity at the carbonyl moiety, it is more readily accommodated by the *endo* approach, leaving the *exo* approach available for the alkyl and chlorine substituents. The same happens for the allocation of the *C*-2 and *C*-3 positions. As the ester is involved in the resonance, it ends up in the first option (*C*-3).

Although it is not sufficiently studied, the change in the solvent’s polarity can have an impact on the resulting product’s asymmetry, even changing the mechanism from a concerted to a stepwise route [93]. The diastereoisomerism arising from reactions with aldehydes can also be reversed by employing secondary amine catalysts [96]. As they attack the carbonyl before the nitrone, making it bulkier, the *endo* configuration is no longer available.

**Scheme 43:**
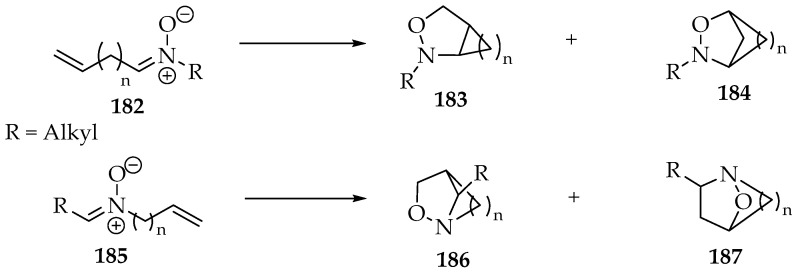
The possible bicyclic products of nitrones’ intramolecular cycloadditions.

For the intramolecular cycloadditions, four types of bicyclic structures can be formed, which are represented in Scheme 43. All of them contain the isoxazolidine fragment. The other carbon ring is bound to different positions for each one. Thus, when the alkene is on the nitrone’s carbon side (Scheme 43, nitrone **182**), its products have the ring bound to the 3,4-positions, compound **183**, and 2,4-positions, compound 184. Whereas, for the alkene in the *N*-substituent (Scheme 43, nitrone 185), the products have the ring bound to the 3,5-positions, compound **186** or 2,5-positions **187**.

The most frequent reactions involve the **182** nitrones, with n = 3 or 4 [3,97]. While the chain is small, the structure **183** is formed. For n = 4, there are already yields of compounds such as **180**. For longer chains, it is expected that a larger ratio of **184**/**183** is required to maintain the same stereoselectivity observed in intermolecular cycloadditions. The case of compounds like **185** (Scheme 43) has not been studied to the same extent, and the products obtained are mostly of the type **187** [98,99]. It is likely that a chain on that side of the nitrone has more freedom than on the other side, since the carbon is in *sp^3^* hybridization.

**Scheme 44:**
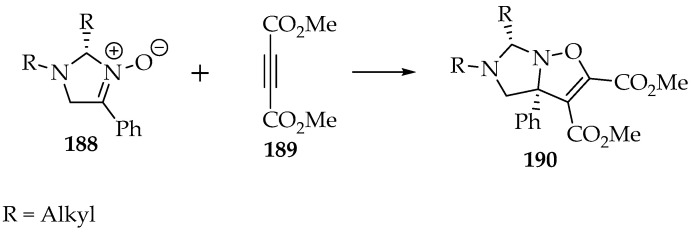
The asymmetric cycloaddition of nitrone **188** to the dimethyl but-2 ynedioate **189**.

Alkynes are more reactive dipolarophiles than alkenes. Therefore, milder conditions are used, often by applying lower temperatures. Also, the heating and the employment of acid or base catalysis can accelerate the rearrangements of the 2,3-dihydroisoxazole product to other compounds [100]. Thus, it should be avoided. The cycloaddition follows a *cis*-configuration and occurs on the least-hindered nitrone’s face (Scheme 44) [101]. The choice of position per substituent in the 2,3-dihydroisoxazole product is similar to the reactions with alkenes [3].

**Scheme 45:**
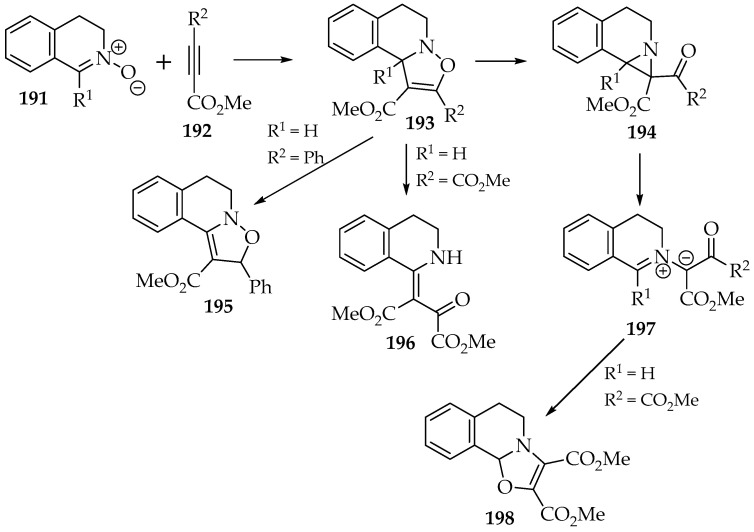
The reactivity of the 2,3 dihydroisoxazoles **193**, resulting from the [3+2] cycloaddition between nitrone **191** and alkynes **192**.

Contrary to the oxazolidines formed in the reaction with alkenes, the 2,3-dihydroisoxazoles **193** are seldom stable. This is even more significant when an aldonitrone 191 is used as a reagent. One possible rearrangement is the 1,3-*H*-shift, which forms the 2,5-dihydroisoxazole **195** (Scheme 45) [100]. This stabilizes the compound once a stronger conjugated *π*-system is established. It becomes more relevant in the presence of aromatic substituents.

Apart from the hydrogen shift, the 2,3-dihydroisoxazole’s rearrangements follow two types of mechanism, with the formation of either the *β*-enamino-ketone **196** or the aziridine **194** (Scheme 45) [100]. They both result from the homolytic (when heated) or heterolytic (acid or base catalysis) cleavage of the *N*—*O* bond. However, the aziridine undergoes a second bond cleavage (*C*—*C*), yielding four possible products. The ylide intermediate **197** might be stable on its own [101]; it can rearrange into an oxazoline **198**.

**Scheme 46:**
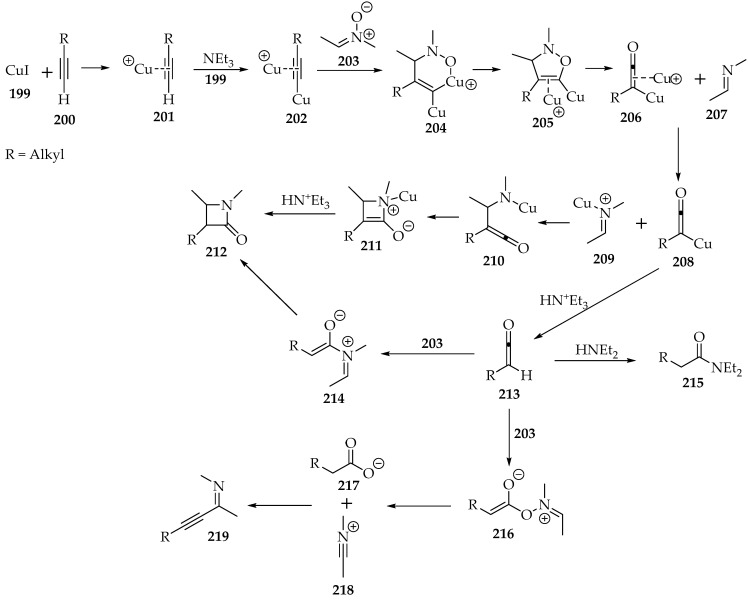
The Kinugasa reaction between a terminal alkyne **200** and the nitrone **199**, catalyzed by copper(I) iodide.

One last type of addition to terminal alkynes **200** is the Kinugasa reaction, which is catalyzed by copper(I) (Scheme 46) [9]. As the alkyne’s hydron is substituted by the Lewis acid (Scheme 46, complex **202**), the resulting 2,3-dihydroisoxazole **205** rearranges to a ketene **210**, and then to an azetidin-2-one **212** (*β*-lactam).

Much discussion has revolved around this reaction’s mechanism. Kinetic studies have shown that the first cyclization to the 2,3-dihydroisoxazole **205** (Scheme 46) is the rate-determining step. It is of the first order on the nitrone and second order on the copper(I) cation, suggesting the dimetalate species [102]. It is supported by theoretical calculations [103]. The alkyne has a negative order of −1, as it consumes the copper required for the reaction catalysis. In essence, the alkyne’s excess forms the complex **201** (Scheme 46), and there is not enough copper(I) for the second complex **202**. The base’s order was not analyzed.

The presence of the metal is detrimental to the cyclization asymmetry. The formation of complex **204** (Scheme 42) reduces the activation energy for that configuration, in contrast to the thermodynamically favored cycloaddition with the R group at position *C*-4 [103].

Then, it was found that the complete fragmentation of the 2,3-dihydroisoxazole **205** (Scheme 46) has a lower activation energy than the partial fragmentation of the *N*-*O* bond only [104]. Thus, the imine **209** and the ketenylcopper(I) **208** are produced, after a metal cation exchange. Afterwards, the *C*-*C* bond is formed, followed by the *N*-*C* bond. The *β*-lactam **212** is obtained after the final hydronation (Scheme 46).

Although it occurs to a lesser extent than the main pathway, ketenylcopper(I) **208** can be hydronated earlier, yielding ketene **213** (Scheme 46). It can still form the intended *β*-lactam **212**, through a Staudinger ketene–imine cycloaddition (Scheme 46, intermediate **214**) [105]. However, the ketene often adds to other nucleophiles. The use of nucleophilic amines should be avoided to reduce the amide side product **215** [102] significantly. In its absence, the nitrone **203** acts as the nucleophile and produces carboxylates **216** and alkynyl imines **219** (Scheme 46), but to a much lesser extent.

In terms of diastereoselectivity, the hydronation of the complex **211** (Scheme 46) happens at the least hindered site, favoring the kinetic *cis*-product [104]. Even if the Staudinger pathway is followed (through compound **214**), and assuming an *exo*-attack of the ketene **213** to the *E*-imine **207**, the same compound should be obtained (Scheme 46) [105]. Nevertheless, it isomerizes to the *trans*-*β*-lactam **212**, which is thermodynamically more stable. The epimerization is considered the responsible mechanism, but the ring opening at the *C*—*C* bond should not be discarded (corresponds to the reverse transformation from **212** to **214**) [106]. Moreover, it is more likely to occur when the *N*-substituent is smaller, or when there is an electron-releasing group at position four and an electron-withdrawing group at position 3 of the *β*-lactam **212** (Scheme 46) [105].

### 3.3. Transformations Fluorinated Nitrones

Nitrones have been identified as effective synthons for the synthesis of a variety of compounds, including heterocycles. While less studied, fluorinated nitrones are particularly noteworthy, as they are gaining recognition as an important synthon for future research and applications.

**Scheme 47:**
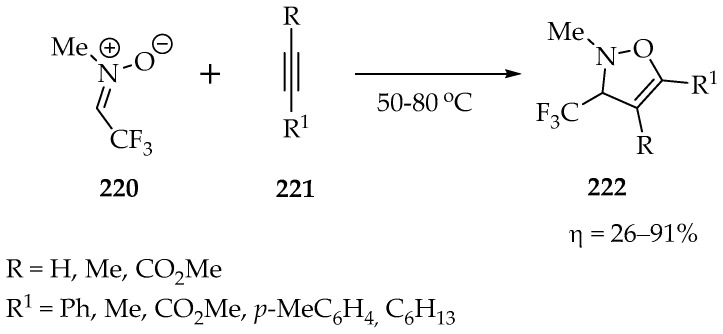
1,3-Dipolar cycloadditions of *N*-methyl-*C*-trifluoromethylnitrone **220**.

One of the first works using fluorinated nitrones was reported by Mitsuhashi et al. [107], who obtained the *N*-methyl-*C*-trifluoromethylnitrone **220** (Scheme 47) via a simple condensation between *N*-methylhydroxylamine hydrochloride and trifluoroacetaldehyde, followed by water removal. Upon obtaining the desired trifluoromethylnitrone **220**, the authors studied its cycloaddition reaction with alkynes and obtained 3-trifluoromethyl-4-isoxazolines **222** (Scheme 47), in low-to-excellent yields, depending on the alkyne substituents (Scheme 47) [107]. The lower yield was obtained with oct-1-yne (26%), and the highest yield was obtained with ethynylbenzene (91%), suggesting that withdrawing groups in the alkyne favored the cycloaddition.

**Scheme 48:**
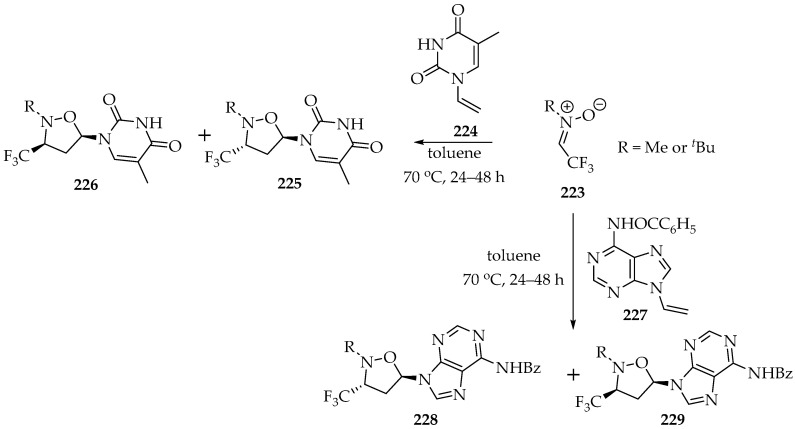
1,3-Dipolar cycloadditions of *N*-alkyl-*C*-trifluoromethylnitrone **223** to *N*-vinyl nucleobases **224** and **227**.

Later on, other researchers used this type of fluoromethylnitrones, changing the N-alkyl group or the number of fluorine atoms [108,109,110], and in doing so they enlarged the scope of derivatives obtained. Moreover, the dipolarophiles were also changed, and alkenes [109,110] or even thioketones [108] were successfully employed. Particularly interesting was the use of *N*-vinyl nucleobases [109] to obtain, in good yields, isoxazolidinyl derivatives of nucleobases (Scheme 48). The yields depend on the nitrone substituent; for example, in the examples shown in Scheme 48, the methyl gives lower yields (38 to 47%), whereas the *t*-buthyl group gives higher yields (54–68%) [109]. Additionally, the methodology employed was proven to have some regio and stereo control, as the dipolarophile geometry is transferred to the cycloadduct. Again, the alkyl groups in the nitrone have some influence, since for the methyl group the major compounds are the *endo*-isomers **225** and **228**, and for the *t*-buthyl group the *exo*-isomers **226** and **229** (Scheme 48) are the major compounds.

**Scheme 49:**
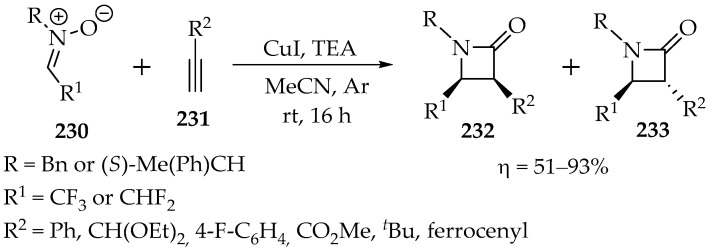
Kinugasa reaction with fluorinated nitrones **230** and acetylenes **231,** leading to fluorinated β-lactams **232** and **233**.

In 2016, Heimgartner et al. published a fascinating study involving the reaction of fluorinated nitrones with alkynes [111]. They demonstrated that these compounds undergo a [3+2]-cycloaddition, followed by transformation into more stable β-lactams, which are obtained in good to very good yields (Scheme 48). While this process results in a diastereomeric mixture of the *cis*-isomer **232** and the *trans*-isomer **233** (Scheme 49), these isomers can be easily separated.

**Scheme 50:**
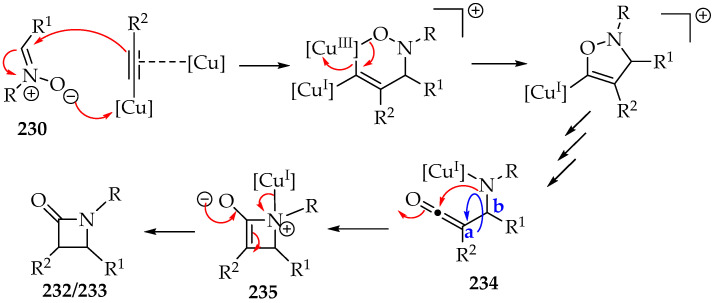
Kinugasa reaction mechanism.

The Kinugasa reaction mechanism (Scheme 50) was recently reviewed [104]. This mechanism explains the production of both diastereomers and demonstrates that bulky substituents on the alkyne lead to higher amounts of the *trans*-isomer **233** (Scheme 49) [111]. Furthermore, the formation of intermediate **234** helps clarify why its cyclization to intermediate **235** may involve rotation around the single bond between carbons **a** and **b** (Scheme 50), yielding a mixture of diastereomers as the final product.

**Scheme 51:**
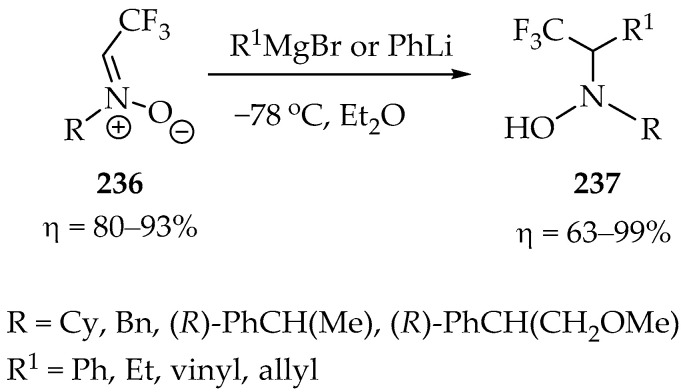
Synthesis of trifluoroethyl *N*-hydroxylamines.

Our last example is an interesting nucleophilic addition of organometallic reagents to trifluoromethyl nitrones **236** (Scheme 51), which were prepared in very good yields from the condensation of various hydroxylamines with fluoral [112]. The synthesis of trifluoroethyl *N*-hydroxylamines **237** was predominantly achieved using Grignard reagents, yielding results ranging from very good to exceptional (Scheme 51). This process not only underscores the remarkable versatility of trifluoromethyl nitrones but also showcases their potential.

In summary, it can be emphasized that these nitrones provide a valuable platform for a range of subsequent synthetic transformations. Due to their intrinsic stability, they present innovative opportunities for the synthesis of biologically active compounds.

## 4. Nitrones’ Applications

Nitrones are already in use or under investigation for future applications. For those purposes, it is helpful to have more insight into their physical–chemical properties.

Nitrones are usually stable at room temperature when kept in the dark. The smaller, aliphatic nitrones are yellowish oils that melt at room temperature, such as 5,5-dimethyl-1-pyrroline *N*-oxide. At the same time, most nitrones contain aromatic groups and have a solid appearance. Their melting points range from 80 to 100 °C for simpler structures, such as *N*,1-diphenylmethanimine oxide [113]. Additional electron-releasing groups or aromatic rings increase that temperature. For example, the *N*-methyl-1-(4-nitrophenyl)methanimine oxide and the *N*,1,1-triphenylmethanimine oxide both melt above 200 °C. However, one should always be concerned with the dimerization possibilities, namely by the [3+3]-cycloaddition mechanism.

The characterization of these compounds is performed by spectroscopic and spectrometric techniques. The ^1^H NMR signal of the proton bound to the sp*^2^*-carbon is shown around *δ* 7 ppm. On the *N*-substituent, the proton signal ranges from δ 3.77 ppm for the methyl to δ 5.89 ppm for the benzyl’s methylene. The ^13^*C* spectra show the sp*^2^*-carbon around *δ* 130 ppm, while the *N*-substituent’s carbon often ranges between *δ* 55 and 80 ppm. The effect of the solvent’s increasing polarity is down shield in the ^1^*H* spectra and slightly up shield in the ^13^*C* [114,115,116,117].

The UV-Vis spectrum varies for non-aromatic and aromatic nitrones. The first case shows a band with a maximum around *λ* 220–250 nm, which corresponds to the π-π* transition [118,119]. The second case shows two bands in the UV-Vis region. The least energetic is around *λ* 300–350 nm, and the most energetic is between *λ* 200 and 250 nm [113,117,120]. They were the subject of discussion in the 60 s [66,119]. The attribution to the nitrone’s π-π* transition and to the aromatic ring’s excitation, respectively, seems the most coherent. Moreover, the first band disappears during photoisomerization to the oxazirane. The spectra’s bands differ slightly between the diastereomers. They are more intense in the (*E*)-1-cyano-*N*,1-diphenylmethanimine oxide than in the *cis* isomer. There is also a hypsochromic shift in the first in comparison to the latter. The presence of substituents that hinder the planarity of the conjugated *π*-system often causes a reduction in the bands’ intensities [113]. The solvent’s polarity and hydrogen bonding also influence the light absorption. More polar solvents decrease the absorptivity, and the maximum wavelength shifts to shorter wavelengths. A hypothesis correlates the hypsochromic shift with the greater polarity of the nitrone relative to its excited state [119,121].

The nitrones’ IR spectra show two characteristic peaks. The one between *λ* 1650 and 1550 cm^−1^ is attributed to the C=N bond and the other, at *λ* 1150–1050 cm^−1^, to the N-O bond [66,114,116,122].

Another important piece of information is the redox potential of this family of compounds. McIntire et al. did cyclic voltammetry in water and acetonitrile, using a calomel reference electrode (SCE) [123]. In the first case, only the reduction potentials were determined, once water was oxidized at +1.2 V, before the nitrones’ oxidation. The reduction potential approximates to −1.8 V for simple nitrones, and the highest value obtained was −0.87 V for an *N*-alkylpyridinium derivative. In the organic solvent, the reduction potential was similar to or lower than that in water, with the difference in the measured potential not exceeding 0.7 V. The anodic potential was the lowest for the sulfonated phenyl nitrone, at 1.34 V. The maximum was obtained for the *N*-alkylpyridinium derivative, with 2.37 V.

Regarding lipophilicity, partition coefficients (*K*P) between 1-octanol and water are determined using HPLC or calculated with appropriate software [37,124,125,126,127]. There is still some variance between results, and the number of experimental works is limited to some nitrones’ derivatives. The two main references are the 5,5-dimethylpiperidine oxide and the *N*-*tert*-butyl-1-phenylmethanimine oxide, whose constants are around 0.1 and 15, respectively [37]. They confirm the good polarity of this organic family. The pyridinyl and phosphoryl substituents increase the hydrophilicity, whereas the bulkier aromatic moieties increase the hydrophobicity. This property is relevant to the toxicity of nitrones. The IC_50_ is often in the millimolar range, but it decreases for more apolar nitrones [124,128]. The related nitroso derivative, obtained upon the nitrone’s oxidative hydrolysis, is slightly more toxic (0.1 mM).

### 4.1. Spin Trap

**Scheme 52:**
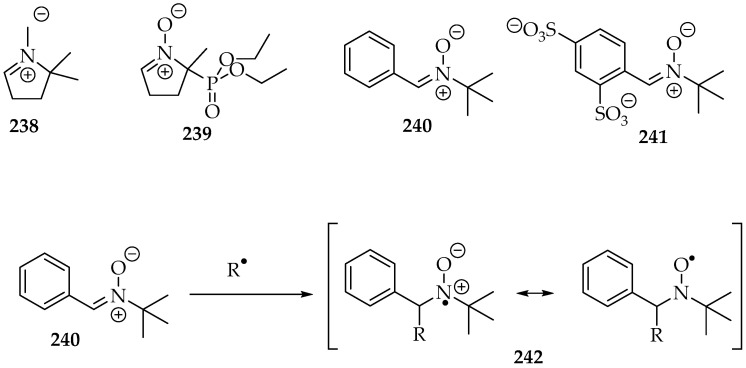
The most used nitrone spin traps: DMPO **238**, DEPMPO **239**, PBN **240,** and disufenton **241**; and the radical R^•^ trapping, with the corresponding resonance stabilization.

Nitrones have been used as spin traps in electron paramagnetic resonance (EPR) experiments, also known as Electron Spin Resonance (ESR). Their goal is to trap radicals produced in specific environments, forming spin-active species (spin adducts), which are then analyzed in the resonance apparatus. Among the nitrone traps, the most ubiquitous are DMPO **238** (2,2-dimethyl-3,4-dihydro-2*H*-pyrrole 1-oxide), DEPMPO **239** (2-(diethoxyphosphoryl)-2-methyl-3,4-dihydro-2*H*-pyrrole 1-oxide), PBN **240** (*N*-*t*-butyl-1-phenylmethanimine oxide), and disufenton **241** (also known as NXY-059; 4-((*t*-butyloxidoazaneylidene)methyl)benzene-1,3-disulfonate), all depicted in Scheme 52 [124]. After the free radical trapping, the nitrone radical formed is stabilized by resonance between the two electronegative atoms (Scheme 52). Moreover, the EPR spectra are characteristic for each free radical with these trapping agents. The signals often show coupling frequencies between the unpaired electron with the nitrogen and the hydrogens of the adjacent carbon, which correlate to specific structures.

**Scheme 53:**
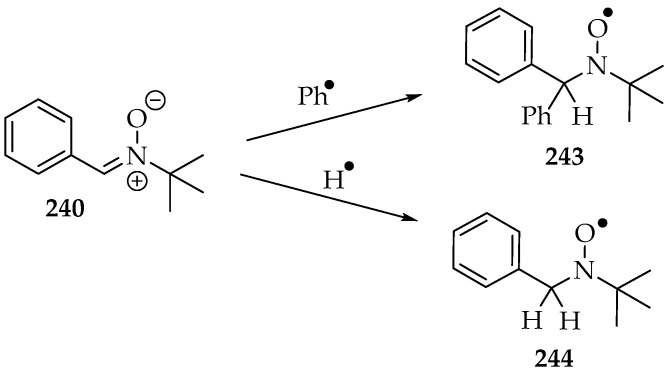
The auto spin adducts formation of water dissolved PBN **240**, under sonication conditions.

However, before considering the trapping of other radicals, it is convenient to understand the radicals that can be produced by the nitrone on its own, especially under sonication conditions. One experiment identified the phenyl radicals from PBN **240** in inert-gas-saturated water solutions (Scheme 53) [129]. The spin adduct **243’s** signal appeared as a double triplet, and its hyperfine coupling frequencies (hfcf) were *aN* = 1.60 mT and *aHβ* = 0.42 mT. The hydrogen radical from water was also identified as a triple triplet: *aN* = 1.67 mT and *aHβ* = 1.05 mT. The hydroxyl radical was too short-lived to be measured. The formation of organic radicals was suggested to occur at gas bubble interfaces, since the organic molecule’s high hydrophobicity favors its presence there. Hence, when the gas bubble bursts, the radicals are produced. That mechanical event is very energetic, far superior to the energy required for the *C*-*C* bond homolytic cleavage.

Another concern in this spin-trapping activity is the formation of artefactual adducts, namely radicals produced by nucleophilic additions rather than by radical scavenging. Two main mechanisms have been described in the literature. They have already been covered before: nucleophile’s addition at the sp^2^-carbon, followed by the hydroxylamine oxidation to the nitroxide radical (either catalyzed by a metal that is reduced in the end, or not); and the initial nitrone’s oxidation, followed by the nucleophile’s attack at the same carbon [124]. The combination of these two pathways is known as the Forrester–Hepburn mechanism. Often, these reactions can be controlled by using chelating agents.

For the radicals to be trapped, not all of them have to have the same affinity for the nitrone. A qualitative scale might be HO^•^ > H_3_C^•^ > HS^•^ > HO_2_^•^ > O_2_^•−^ > NO [124]. Some strategies can be envisioned to improve them, namely for the superoxide radical anion (O_2_^•−^). It can be protonated at lower p*H*, forming the slightly stronger HO_2_^•^ radical. Including hydrogen-donating groups for hydrogen bonding, such as amides and cyclodextrins, is another possibility. For the weakest species of the list, nitric oxide (NO), other trapping agents are used, rather than the nitrones.

In practical experiments, the concentrations of nitrone spin traps range from 50 to 100 mM, so that the signal is detectable by ESR [115]. Considering biological applications, these high concentrations are a relevant drawback. Nevertheless, research has been conducted to improve the affinity of nitrones to the biological radicals in general, so that those concentrations can be lowered. For example, the combination of EMPO [2-(ethoxycarbonyl)-2-methyl-3,4-dihydro-2*H*-pyrrole-1-oxide] with gold nanoparticles enabled a 370-fold decrease in the concentration requirements, reducing from 10 mM for EMPO to 27 μM for Au@EMPO.

### 4.2. Therapeutic

One of the most important applications is the extensive study of nitrones for medical applications, as well as for the detection of biological radicals by ESR. Their own action against the reactive radicals, such as ROS and RNS (Reactive Oxygen and Nitrogen Species, respectively), by scavenging them, is already relevant in controlling the cells’ oxidative stress. Moreover, many of those adducts’ degradation products have beneficial properties. The degradation pathway accounts for the hydrogen on the *sp*^2^-carbon, which makes it much more reactive [124]. Furthermore, due to this degradation mechanism, the radical scavenging is irreversible.

The degradation of the nitrone-superoxide adduct results mainly in the hydrogen peroxide (H_2_O_2_), along with the nitric oxide (NO) and the hydroxamic acids (*N*-hydroxyamides) products. The peroxide production resembles the biological superoxide dismutase (SOD) enzyme and yields higher amounts than the other two products. Nitric oxide is a well-known signaling agent, a vasodilator, and a molecule involved in the immune response, by targeting iron–sulfur centers and damaging the DNA of parasites [130]. Nitrones are considered NO slow-releasers, as the superoxide disproportionation is faster than the former, which is even inhibited in alkaline media [131]. Finally, the hydroxamates are good metal ligands, especially for iron. Therefore, they can prevent iron-catalyzed Fenton reactions.

When trapping is performed with carbon radicals, the resulting nitroxide can be disproportionate to the nitrone and the hydroxylamine (Scheme 3A). That hydroxylamine further enhances the redox activity of such therapeutic agents [124,132].

Research into nitrones as pharmaceuticals began in 1986 with the work of Novelli et al. [133]. The study focused on the prevention capacity of PBN in rats that were subjected to a lethal body trauma, using a rotating drum. It was reported to be highly effective [133]. Since then, nitrones have been applied in the treatment of many diseases, such as cancer, stroke, Parkinson’s, Alzheimer’s, and aging, and have been recognized as potent anti-inflammatories [133]. The most promising compound so far, which entered phase 3 clinical trials for stroke under the AstraZeneca brand, is NXY-059 **241** (Scheme 52) [134]. Unfortunately, no significant improvement in the patients’ treatment was observed in the last trial, in 2006. One reason might be the reported non-specificity for intracellular targets [124].

Nitrones’ partition coefficient varies with the bound groups, being 30 for PBN **240** (Scheme 52) and below 1 for more hydrophilic ones [124]. A solution to improve the substance’s retention within cells is to esterify it with the acetoxymethyl group. Other alternatives, such as binding the nitrone to specific compounds and receptors, have also been tested. One such example is the triphenylphosphonium lipophilic cation derivative, which was shown to concentrate the nitrone inside mitochondria. Other examples are amphiphilic, glycosylated, and peptidic molecules. Commonly used linkers are amides, esters, and phosphoryls. The IC_50_ for this family of organic molecules is usually in the millimolar range, with the more lipophilic ones having lower values [124]. For example, DMPO **238** (Scheme 52) is less toxic (IC_50_ 140 mM) than PBN **240** (IC_50_ 9 mM) in bovine aortic endothelial cells.

Despite the critical nature of this application and the emphasis on its importance, considerable effort is still needed to fully explore the therapeutic potential of nitrones. The journey ahead holds great promise and invites further investigation.

## 5. Conclusions

In summary, this comprehensive review highlights the critical role of nitrones in organic chemistry. It covers their synthetic methodologies, diverse applications in organic synthesis, physicochemical properties, potential therapeutic relevance, and opportunities for spin trapping.

The versatility of reactions involving nitrones highlights the need for further exploration of their synthesis. Some known methodologies for preparing nitrones have been thoroughly elucidated and explained, utilizing the best available knowledge. However, some mechanisms still require confirmation, and the conversion rates of specific reactions are not always complete. So, in-depth mechanistic studies can provide valuable insights into the fundamental principles governing these reactions and pave the way for the development of more efficient and selective processes. Additionally, the emergence of new metal-catalyzed reactions has opened up exciting possibilities in chemical synthesis and ensures that there remains ample room for improvement and discoveries.

Nitrones’ ability to scavenge free radicals and reduce oxidative stress in biological systems presents promising opportunities for the development of new therapeutic agents. Investigating nitrones as potential treatments for various diseases, including neurodegenerative disorders and cardiovascular conditions, is a promising area for future research.

In industry, nitrones can be explored in radical polymerization, enabling the production of advanced polymeric materials with new molecular structures that can be efficiently designed. The extent of their impact on fields such as nanotechnology, optics, and supramolecular chemistry remains to be fully explored.

Ultimately, this review of nitrones aims to reveal their chemical properties, stimulate the development of novel reactions, facilitate the discovery of potential therapeutics, and broaden their applications across various fields.

## Abbreviations

The following abbreviations are used in this manuscript:

Ac	Acetyl
AIBN	2,2′-Azobis(2-methylpropionitrile)
Ar	Aryl
BINAP	([1,1′-Binaphthalene]-2,2′ diyl)bis(diphenylphosphane)
Bn	Benzyl
BPO	Benzoyl peroxide
bpy	2,2’-Bipyridine
Bu	Butyl
Cp*	1,2,3,4,5-Pentamethylcyclopentadienyl
DCE	1,2-Dichloroethane
DCM	Dichloromethane
DEPMPO	2-(Diethoxyphosphoryl)-2-methyl-3,4-dihydro-2*H*-pyrrole-1-oxide
DIBAL	Diisobutylaluminium hydride
DIPEA	*N*-Ethyl-*N*-(propan-2-yl)propan-2-amine
DMD	Dimethyldioxirane
DMPO	2,2-Dimethyl-3,4-dihydro-2*H*-pyrrole 1-oxide
DNA	Deoxyribonucleic acid
E/E^+^	Electrophile
EMPO	2-(Ethoxycarbonyl)-2-methyl-3,4-dihydro-2*H*-pyrrole-1-oxide
ESR	Electron spin resonance
EPR	Electron paramagnetic resonance
Et	Ethyl
Hfcf	Hyperfine coupling frequency
HPLC	High performance liquid chromatography
IBX	*o*-Iodoxybenzoic acid
IPr	1,3-Bis(2,5-diisopropylphenyl)imidazol-2-ylidene
IR	Infrared
IUPAC	International Union of Pure and Applied Chemistry
LDA	Lithium diisopropylamide
LTMP	Lithium tetramethylpiperidide
*m*-CPBA	3-Chlorobenzene-1-carboperoxoic acid
Me	Methyl
Mes-Acr^+^	9-Mesityl-10-methylacrydinium
MIP	2-Methoxyisopropyl
MTO	Methylrhenium trioxide
Ms	Mesyl/methylsulfonyl
MS	Molecular sieves
NBS	1-Bromopyrrolidine-2,5-dione
NMR	Nuclear magnetic resonance
Nu/Nu^−^	Nucleophile
Oxone^®^	Potassium peroxysulfate
PBA	Perbenzoic acid
PBN	*N*-*t*-Butyl-1-phenylmethanimine oxide
Ph	Phenyl
Phen	1,10-Phenanthroline
PIN	Preferred IUPAC Name
PPTS	Pyridinium *p*-toluenesulfonate
ROS	Reactive Oxygen Species
RNS	Reactive Nitrogen Species
SCE	Saturated calomel electrode (0.241 V vs. SHE, 25 °C)
SHE	Standard hydrogen electrode
SOD	Superoxide dismutase
TBAF	Tetrabutylammonium fluoride
TBDMS	*tert*-Butyldimethylsilyl
TBDPS	*tert*-Butyldiphenylsilyl
TEA	Triethylamine
Tf	Triflyl
TFA	Trifluoroacetic acid
THF	Tetrahydrofuran
TIPS-EBX	1-[(Triisopropylsilyl)ethynyl]-1,2-benziodoxol-3-(1*H*)-one
TMS	Trimethylsilyl
Tol	Tolyl/(methylphenyl)
Ts	Tosyl/((4-methylphenyl)sulfonyl)
